# Comparative phylogeography in the Atlantic forest and Brazilian savannas: pleistocene fluctuations and dispersal shape spatial patterns in two bumblebees

**DOI:** 10.1186/s12862-016-0803-0

**Published:** 2016-12-07

**Authors:** Elaine Françoso, Alexandre Rizzo Zuntini, Ana Carolina Carnaval, Maria Cristina Arias

**Affiliations:** 1 Instituto de Biociências, Universidade de São Paulo, Rua do Matão, 277, sala 320, 05508-090 São Paulo, SP Brazil; 2 Instituto de Biologia, Universidade Estadual de Campinas, Rua Monteiro Lobato, 255, 13083-970 Campinas, SP Brazil; 3 Department of Biology, City College of New York, New York, USA; 4 The Graduate Center, City University of New York, New York, NY USA

**Keywords:** Comparative phylogeography, Bumblebee, Brazil, mtDNA, Microsatellites, Geographic distribution modeling

## Abstract

**Background:**

*Bombus morio* and *B. pauloensis* are sympatric widespread bumblebee species that occupy two major Brazilian biomes, the Atlantic forest and the savannas of the Cerrado. Differences in dispersion capacity, which is greater in *B. morio*, likely influence their phylogeographic patterns. This study asks which processes best explain the patterns of genetic variation observed in *B. morio* and *B. pauloensis*, shedding light on the phenomena that shaped the range of local populations and the spatial distribution of intra-specific lineages.

**Results:**

Results suggest that Pleistocene climatic oscillations directly influenced the population structure of both species. Correlative species distribution models predict that the warmer conditions of the Last Interglacial contributed to population contraction, while demographic expansion happened during the Last Glacial Maximum. These results are consistent with physiological data suggesting that bumblebees are well adapted to colder conditions. Intra-specific mitochondrial genealogies are not congruent between the two species, which may be explained by their documented differences in dispersal ability.

**Conclusions:**

While populations of the high-dispersal *B. morio* are morphologically and genetically homogeneous across the species range, *B. pauloensis* encompasses multiple (three) mitochondrial lineages, and show clear genetic, geographic, and morphological differences. Because the lineages of *B. pauloensis* are currently exposed to distinct climatic conditions (and elevations), parapatric diversification may occur within this taxon. The eastern portion of the state of São Paulo, the most urbanized area in Brazil, represents the center of genetic diversity for *B. pauloensis.*

**Electronic supplementary material:**

The online version of this article (doi:10.1186/s12862-016-0803-0) contains supplementary material, which is available to authorized users.

## Background

Although the field of comparative phylogeography emerged from studies of the role of common landscape or climatic shifts on the distribution of genetic diversity across sympatric species [1, 2], it has become clear that phylogeographic structure and its underlying drivers are not necessarily shared among all members of a community [3]. Lack of topological congruence across gene genealogies of co-occurring species has been observed whenever they differ in ecology, past ranges, impending selective pressures, mutation rates, effective population sizes, local extinction rates, and dispersal ability, or due to the implicit randomness of gene coalescence [4–16]. We explore how ecological differences between two widespread species of *Bombus* bees co-distributed along most of eastern South America impact their levels and patterns of diversity. *Bombus* is a genus of pollinators of vital importance for natural ecosystems and mankind. It is typically Holartic and finely adapted to cold climate, showing a higher number of species and subgenera in the Palearctic relative to the Nearctic and Neotropic regions [17, 18]. These robust and hairy bees have thermoregulatory adaptations involving facultative endothermy [19], which enables them to inhabit high altitudes and cold temperatures. Among the few species found in the Neotropics are *B. morio* and *B. pauloensis*, which occur in sympatry over a large area in Brazil. These bees are mainly found in high elevation areas from the state of Rio Grande do Sul to the states of Minas Gerais and Espírito Santo [20], occupying two Brazilian diversity hotspots: the Atlantic forest and the Cerrado savannas.

Depending on the classification system used these two species are considered as entities belonging to the same subgenus *Fervidobombus* [18] or to different ones, the latter and *Thoracobombus* [21]. *Bombus morio* and *B. pauloensis* behave similarly, have nearly the same geographical distribution, and ecological and trophic niches [22, 23]. Yet they differ in their morphology and inferred ability to disperse. *Bombus morio* is thought to have higher dispersion capacity: its coloration is uniformly black, and the species has a more robust size compared to the other Brazilian bumblebees, which allows for longer flight time [20]. This species also seems to have a preference for forest habitats, being more commonly observed in gallery forests, which, according to Moure & Sakagami [20], may further increase its dispersal. *Bombus pauloensis,* on the other hand, is the most polytypic Brazilian species, and known for its high level of intra-specific variation in body color and habitat [20].

Although the distribution of both species of *Bombus* would extend beyond Brazilian frontiers, the ranges of both *B. morio* and *B. pauloensis* in Brazil are centered in the state of São Paulo, a complex region where phylogeographic breaks have been reported in species of amphibians [24–27], bats [28], birds [29], and snakes [30]. Multiple processes have been loosely associated with and suggested to underline these patterns, including persistence in isolated Pleistocene refugia [24, 28, 29, 31, 32], differentiation across river barriers [33], and vicariance through tectonic movements [25–27, 34]. We investigate whether spatial patterns of genetic diversity within *B. morio* and *B. pauloensis* support these hypotheses while taking their ecological differences in consideration. Particularly, we focus on the documented differential dispersal abilities and physiological tolerances of these two species and ask i) whether their differential dispersal abilities are tied to distinct infraspecific tree topologies and historical demography (where topographical incongruence and less genetic structure is expected for the high dispersal *B. morio*), and ii) whether tolerance to cooler environments allowed these species to expand their ranges in the Last Glacial Maximum (LGM), as shown by genetic signatures of expansion toward the north, contrasting with range contraction in the subsequent interglacial period. If evidence suggests that these species have tracked the cooler, more sub-tropical conditions during the Late Quaternary, these data will be in sharp contrast with the abundant examples emerging from studies of Atlantic forest lowland taxa [24, 28, 29, 31, 32]. We here test whether *Bombus* bees may be pinpointed as models of cold-associated forest species in studies of responses to climate change in eastern South America over the past hundred thousand years.

## Methods

### Sampling

**Fig. 1:**
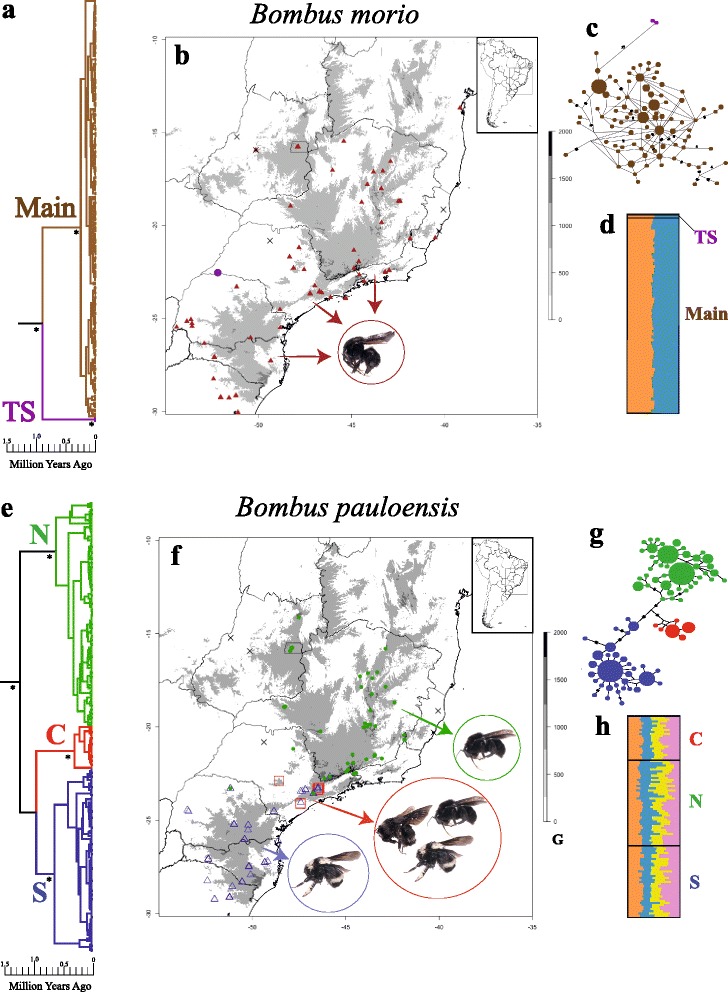
Phylogeographic lineages found in *Bombus morio* (183 samples) and *B. pauloensis* (221 samples) from 1570 bp of mitochondrial DNA (C*ytocrome C oxidase I*, *Cytochrome B*, the large ribosomal RNA subunit, and cluster 4 of tRNA, covering a region of *COII* and *ATPase 8* genes and tRNA^lys^ and tRNA^asp^). **a**, **e** coalescent bayesian phylogeny calibrated with fossils according to Hines [39] in the *Thoracobombus* node, that includes *B. morio* and *B. pauloensis*, dated to 13,5962 Ma (min. 7,6283 Ma, max. 21,5374 Ma). *Bombus pauloensis* was used as outgroup for the *B. morio* matrix and vice versa. Asterisks indicate clades with high support of posterior probabilities (>0.97). **b**, **f** sampling localities with the respective color found in the phylogenetic cluster. Grey areas in the map correspond to altitudes above 750 m, “X” represents missing data. **c**, **g** haplotypes networks. The size of the nodes is proportional to frequencies. Traces on the line correspond to mutational steps, and the absence of trace corresponds to one mutational step. **d**, **h** groups found in Structure software, according to microsatellite data, correlated with the clusters found in mitochondrial data. Main: main clade; TS: Teodoro Sampaio clade; C: central clade; N: north clade; S: south clade

A total of 183 individuals of *B. morio* and 221 *B. pauloensis* was obtained during field trips and from museum collections, covering the greater part of the total distribution in Brazil (Fig. 1b and f; Additional file 1 for voucher numbers, species name, locality, year, collector, tissue conservation method, latitude, and longitude). Although Moure’s bee catalogue (http://moure.cria.org.br) provides a larger range of distribution for both species, we considered these distributions inaccurate and overestimated, since presumably occurrence sites and local collections were visited and no bumblebee were found in the last decades. Specimens were identified according to the morphological key proposed by Moure & Sakagami [20]. Despite collecting efforts in different periods of the year and visits to local collections, we were unable to find samples in northern Espírito Santo and northern São Paulo (Fig. 1b and f). In western Goiás, only a single queen of *B. morio* was found.

DNA extraction was performed by the Chelex® 100 method (BioRad, United Kingdom). It included the use of one middle leg of frozen specimen in 400 ul of 10% Chelex, mechanical maceration, incubation at 56 °C for 30 min, vortex for 10 s, incubation at 100 °C for 5 min, vortex for 10 s, and centrifugation for 1 min at 14,000 rpm. The supernatant was used for PCR amplification. A DNeasy Tissue Kit (Qiagen, Germany) was used to extract DNA from pinned specimens according to the supplier’s recommendations.

### Molecular sampling

#### Mitochondrial DNA data

We partially sequenced the following mitochondrial markers: *Cytochrome C oxidase I* (*COI*), *Cytochrome B* (*CytB*), the large ribosomal RNA subunit (*16S*), and cluster 4 of tRNAs (Cl4, encompassing the *COII* 3’ region, tRNA^lys^, tRNA^asp^, and the *ATPase 8* 5’ end; see Table 1 for primers). Polymerase Chain Reactions (PCRs) were set up with 2.0 μl of DNA template in a 20 μl final volume containing 1x PCR buffer, 0.4 μM each primer, 0.2 mM each dNTP, 1.5 mM MgCl_2_, 1.5 U of Taq DNA polymerase (Invitrogen, USA), and 1 M betaine (USB, USA). Reactions were performed in a Mastercycler Pro (Eppendorf, Germany) and consisted of an initial denaturation step at 94 °C for 5 min followed by 35 cycles at 94 °C for 1 min, 42 °C for 80 s, and 64 °C for 2 min, and a final extension at 64 °C for 10 min. PCR products were separated on a 0.8% agarose gel, stained with Gel Red 10,000X (Biotium, USA), and visualized under UV light. The PCR products were purified with 0.5 μl of ExoSAP-IT® (USB, USA) following the thermal treatment recommended by the manufacturer, and sequenced by Macrogen (South Korea). PCR primers were used for sequencing. The program Muscle [35] included in Geneious Pro 7.0.6 software (available from www.geneious.com) was used to align the sequences. The phred quality score in Geneious was used to represent an estimate of error probability in the sequencing. Q20, Q30, and Q40 indicate error probabilities of 1 in 100 (10^2^), 1,000 (10^3^) or 10,000 (10^4^), respectively. The *COI* and *CytB* sequences were edited and translated into amino acid sequences to ensure that no nuclear mitochondrial DNA (NUMT) misamplification had taken place.

**Table 1:** Characteristics of the mitochondrial regions analyzed for *Bombus morio* (Bm) and *B. pauloensis* (Bp)

Mitochondrial region	sp.	Sequence size (bp)	Variable sites	Frequency (%)				h	Hd	Model	Primers pairs
				A	C	G	T				
*COI*	Bm	399	26 (6.5%)	30.6	13.6	10.2	45.6	26	0.7607	TPM1uf + I + G	mtD6 and mtD9 [103]
	Bp	399	17 (4.3%)	30.8	13.6	10.4	45.3	25	0.7846	TIM1 + I	
*CytB*	Bm	396	24 (6.1%)	34.9	12.8	7.6	44.7	33	0.8686	GTR + G	AMB16 [104] and mtD26 [103]
	Bp	396	21 (5.3%)	30.0	12.8	6.2	43.0	26	0.8202	HKY + I	
*16S*	Bm	378	10 (2.6%)	42.0	6.9	11.7	39.4	11	0.2709	HKY + I	Cox2 and Atp8rev [105]
	Bp	378	7 (1.9%)	40.2	7.5	11.7	40.7	8	0.1388	TPM1uf	
*Cl4*	Bm	397	24 (6%)	40.2	9.8	11.0	39.0	22	0.6735	TrN + I	16SF and 16SR [103]
	Bp	396	24 (6.1%)	40.7	10.4	9.9	39.1	29	0.8850	TrN + G	
Concatenated	Bm	1570	86 (5.4%)	36.8	10.8	10.1	42.2	94	0.9677	-	-
	Bp	1569	71 (4.5%)	37.3	11.1	9.5	42.1	72	0.9501	-	-

*COI: Cytochrome C oxidase I*; *CytB: Cytochrome B*; *16S*: large ribosomal RNA subunit; Cl4: cluster 4 of tRNA (covering a region of *COII* and *ATPase 8* genes and tRNA^lys^ and tRNA^asp^); sp.: species; h: number of haplotypes; Hd: haplotype diversity; Model: nucleotide substitution model

#### Heteroplasmic loci

The *COI* and *CytB* electropherograms of *B. morio* presented two peaks across multiple sites in the sequencing, indicating heteroplasmy by absence of stop codons and frame shift mutations. Two tests were performed to verify if these double peaks had a direct influence on the topology. First, we excluded all sites of multiple peaks from the analysis, and estimated the topology. In addition, we recovered all possible haplotypes with a probability of 90% and used them in a second topology estimation. To this end, all ambiguous sites were scored with IUPAC ambiguity codes, thus including double peaks in a non-conservative way. Mitochondrial haplotypes were separated using seqPHASE [36] for each sample. The data set of inferred haplotypes is hereafter referred to as “phased data set.”

#### Nuclear microsatellite data

Sixty-seven specimens of *B. morio* and 96 of *B. pauloensis* were screened for the eight most polymorphic loci previously determined for each species [37, 38] (Additional file 1).

### Dating and coalescent-based inferences

To provide calibration points for our subsequent analyses, we used DNA sequences, fossils, and tree data from Hines [39], and manually added three sequences of *B. morio* (specimens 30, 177, 211) and two of *B. pauloensis* (176, 178) to the data matrix available in treeBASE (Study ID: S1927; http://www.treebase.org) [39]. For each sample, we generated DNA sequences of three of the five molecular markers used by Hines [39]: 16S, ArgK [40], and EF-1α [41], using primers and conditions specified in those respective publications. To estimate the age of the root of *Bombus* and of the nodes of our samples, we applied the same models of nucleotide substitution and calibration points used by Hines [39]: 44.1 Ma for *Liotrigona mahafalya*-*Hypotrigona gribodoi*, 15−20 Ma for *Plebeia frontalis*-*Trigona amazonensis*, and 80–100 Ma for Meliponini-Bombini. The tree available in treeBASE was used as a starting tree, adding our five specimens as sister clades to the respective species sampled in Hines [39]. For each marker we used independent partition and molecular clock. Markov chain Monte Carlo (MCMC) analysis was performed under a Yule speciation process model, with a lognormal relaxed clock [42], through 20 million generations. The prior for the mean of each rate was set to 1, on a normal distribution. The analysis was performed in Beast v1.8.0 [43]. Run quality was conferred in Tracer v1.6 with a threshold of 100 for the effective sample sizes (ESSs). The final tree was summarized in TreeAnnotator, following a 20% burn in, and was visualized and edited in FigTree v1.4.0 (http://tree.bio.ed.ac.uk/software/figtree/). As expected, the final tree showed branch lengths very similar to that of Hines [39], and the *Bombus* root was dated at ~34 Ma. We used the node corresponding to the most recent common ancestor of *B. morio* and *B. pauloensis* (dated at 13.5962 Ma ±3; min. 7.6283, max. 21.5374; Additional file 2) as a calibrated node to guide dating analyses. This node is also especially relevant because of its high posterior probability value (100%).

A calibrated mtDNA genealogy was inferred for each species separately, including the phased data of *B. morio. Bombus pauloensis* (sample USP2) was used as outgroup for the *B. morio* inference, and *B. morio* (sample USP1) was used as outgroup for *B. pauloensis*. Selection of the best-fit nucleotide substitution models for each species and mitochondrial region was made in JModelTest 2.1.4 [44], using the Akaike information criterion (Table 1). Markov chain Monte Carlo (MCMC) analyses were performed with a coalescent, constant size tree prior, and applying a lognormal relaxed clock. Trees were ran for 40 million generations, and sampled every 1,000 generations.

### Genetic diversity and structure

#### Mitochondrial data

Numbers of haplotypes, haplotype distribution, variable sites, average number of nucleotide differences (k), haplotype diversity (Hd) and nucleotide diversity (π) were calculated in DnaSP 5.10.1 [45]. Haplotype networks were constructed in Network 4.6.1.1 (www.fluxus-engineering.com) using the median joining algorithm [46]. Tajima’s D, Fu's Fs, and Fay statistics tests for population expansion, as well F_st_ values, were obtained in Arlequin v.3.5 [47]. Genetic distance between major mitochondrial lineages of *B. morio* was inferred through the sum of the length of branches of a UPGMA tree obtained in Geneious Pro software.

#### Nuclear microsatellite data

We created a matrix of microsatellite data for *B. morio* and *B. pauloensis* (Additional files 3 and 4) and assessed genetic diversity using allele frequencies. Expected heterozygosity (He), observed heterozygosity (Ho), and F_st_ values were calculated on GeneAlex v. 6.5 [48]. Allelic richness (based on minimum sample size) and inbreeding coefficient (F_IS_) were calculated with FSTAT 2.9.3.2 [49]. Tests for Hardy–Weinberg equilibrium (HWE) were performed in GENEPOP 4.1 [50]. Homozygote excess for each locus was verified with MICRO-CHECKER 2.2.3 [51]. Since the microsatellite primers used here are species-specific and were previously tested in a HWE population under exactly the same amplification conditions [37, 38], we expect that the homozygote excess will reflect the population structure, not the presence of null alleles. To classify individuals into genetic groups (K) and estimate the proportion of genetic mixing of each individual (Q), we used the Bayesian attribution method implemented in Structure 2.3.3 [52]. The data were analyzed using different values of K (1–10), without considering the origin (which allows for finding structure inside the population) in order to determine the most likely number of groups. We ran 1,000,000 MCMC replicates with a 100,000 burn-in, and 20 interactions for K, to ensure statistical stability [52]. We used an admixture model (each individual draws some fraction of its genome from each of the K populations) and correlated allele frequencies [53]. Simulation results were converted into graphs for visual analysis using Structure Harvester [54]. The 20 interactions were aligned in CLUMPP 1.1.2 [55], and graphical results were displayed in DISTRUCT 1.1 [56]. The best K value was estimated using delta K, based on the second order rate of change of the likelihood scores [57].

### Geographic distribution modeling

We used Maxent v.3.3.3 k [58, 59] with default settings and a subset of 10% of the points for model testing to generate correlative species distribution models under present and past climatic conditions, based on bioclimatic variables from the WorldClim data set [60]. Maxent generates models using presence-only records, contrasting them with pseudo-absence or background data resampled from the remainder of the study area.

For input locality data, we used 102 and 71 different georeferenced occurrence records for *B. morio* and *B. pauloensis*, respectively, from our samples (Additional file 5). For each species, we developed present-day models (5 km resolution) and then projected them to the Last Interglacial (LIG, ~120,000–140,000 years BP) [61] and Last Glacial Maximum (~21,000 years BP) conditions. For the LGM, we used stacked projections of MIROC and CCSM models and used the minimum value of each cell. We used this approach to identify the most suitable areas in both projections, since the projection sum overestimates the area and the projection intersection underestimates the area. We modeled averages of ten replicates using the “crossvalidate” option. Model performance was evaluated using the Area Under the Curve (AUC) calculated by Maxent. AUCs > 0.75 are typically considered adequate for species distribution modeling applications [62].

### Environmental influence in the species distribution analysis

We used a Principal Component Analysis (PCA) to examine the variance and correlation across the 19 environmental measurements (according to WorldClim) along the sampled range of *B. morio* and *B. pauloensis*. For that, we plotted the environmental conditions at a single point per grid cell per mitochondrial lineage for each species among all our samples. Climates were characterized according to the Köeppen-Geiger climatic classification [63].

As the two species seem to mainly occupy regions of higher elevations (Fig. 1b and f), the influence of altitude was verified by extracting altitude values for our sampling localities. Each locality was considered just once by clade in *B. morio* and *B. pauloensis*. Abundance was not considered since we cannot affirm that the collection efforts were the same in every locality. GTOPO 30 was used as the digital elevation model, available from the U.S. Geological Survey (https://lta.cr.usgs.gov/GTOPO30).

All maps were made in R [64], using Raster [65] and Maptools [66] packages.

## Results

### Coalescent phylogeny, molecular clock analyses, and haplotype networks

For each mitochondrial region analyzed in this study, the following information was recorded: size of fragments sequenced, variable sites, nucleotide frequency, number of haplotypes, and haplotype diversity (Table 1). Bayesian phylogenies of *B. morio* and *B. pauloensis,* based on the mtDNA data, show different topologies (Fig. 1a and e). Most *B. morio* samples are clustered in a well-supported clade with no internal structure; a second well-supported clade encompasses two samples, both from Teodoro Sampaio, a town located in the western portion of the state of São Paulo (Fig. 1a and b). Genetic distance between these clades is 1.73%. *Bombus pauloensis*, in turn, show three distinct and strongly supported clades (posterior probabilities > 0.97) but weakly supported basal nodes, basically forming a trichotomy due to those low posterior probability values. The three clades are here named according to their geographic extent: central (C clade), northern (N clade), and southern (S clade). None of these clades present well supported internal structure (Fig. 1e and f). Dating suggests that the major splits in the gene trees occurred between 100,000−200,000 years ago (Fig. 1e and f).

Haplotype networks allow the visualization of the same general inferences provided by the phylogenetic analyses. *Bombus morio* shows differentiation between Teodoro Sampaio samples and all other individuals from throughout the range of the species, and no genetic structure within the latter. Reticulations are conspicuous in this network (Fig. 1c). This species showed double peaks throughout its sequences, and the reticulation is observed even when the sites with multiple peaks were excluded. The most abundant haplotype was verified in 26 samples and was broadly distributed from south to north and east to west, sometimes present in locations 2,000 km apart (e.g., Lajeado in the state of Rio Grande do Sul to Igrapiúna in the state of Bahia).

The haplotype network of *B. pauloensis* allows the visualization of clades C, N, and S, as observed in the phylogenetic analysis. Haplotypes differ by few mutation steps (Fig. 1g). In the N clade, the two most distant sites are located ~1,000 km apart (from Itatiaia in the state of Rio de Janeiro to Alto Paraíso de Goiás, in the state of Goiás); in the S clade they are, maximally, ca. 800 km apart (from Caxias do Sul in the state of Rio Grande do Sul to São Paulo, in the state of São Paulo).

Bees of the three mitochondrial clades of *B. pauloensis* differ in frequency of body color patterns (Fig. 1f). The S clade encompasses bees with regular yellow stripes. The N clade encompasses bees with the whole body black, with few exceptions. The C clade encompasses bees with the whole body black and also bees with irregularly spaced yellow stripes.

In *B. morio*, at least 3% of *COI* and 1.75% of *CytB* sequences sites showed double peaks and low *phred* score values (< Q20; error probabilities of 1 in 100), despite the absence of stop codons and frame shifts (Additional file 6). Sequencing was repeated for selected individuals to ensure that the results were not due to PCR contamination, and the same double peaks were consistently found. Double peaks were not related to specific individuals or geographic regions. Exclusion of these loci from downstream analyses resulted in unchanged phylogeny and haplotype network topologies. The phased data set consisted of 366 sequences (from 183 individuals of *B. morio*), and the calibrated Bayesian phylogeny was congruent with that inferred with the original, unphased, dataset (two structured clades with high posterior probabilities, lack of structure within clades; data not shown). Double peaks were not observed in *B. pauloensis*.

### Genetic diversity and structure

#### Mitochondrial data

*Bombus morio* and *B. pauloensis* show high levels of Hd yet low π. Fu`s Fs and Tajima`s D statistics showed significant negative values (Table 2). In *B. morio*, F_st_ values between the two main mitochondrial lineages was high (0.82429), indicating strong differentiation between the Teodoro Sampaio and Main clades (Table 3). F_st_ values obtained across the C, N, and S lineages of *B. pauloensis* were also very high (0.79 N vs. S; 0.78 C vs. S; 0.76 C vs. N).

**Table 2:** Genetic diversity indices for each clade of *Bombus morio* (Main: main clade; TS: Teodoro Sampaio clade) and *B. pauloensis* (N: north clade; C: central clade; S: south clade) obtained from 1,575 bp of the following concatenated mitochondrial regions: *Cytochrome C oxidase I* (*COI*), *Cytochrome B* (*CytB*), the large ribosomal RNA subunit (*16S*), and cluster 4 of tRNA (covering a region of *COII* and *ATPase 8* genes and tRNA^lys^ and tRNA^asp^)

	*B. morio*			*B. pauloensis*			
	Main	TS	All	C	N	S	All
Ns	181	2	183	21	110	90	221
h	87	2	89	6	36	30	78
Hd	0.963	-	0.964	0.72381	0.91009	0.87491	0.95483
*k*	4.349	-	4.92374	1.20952	3.30525	2.89588	8.378
π	0.00278	-	0.00315	0.00077	0.00184	0.00170	0.00537
Tajima`s D (*p*-value)	-1.85387 (0.00600)	0.00000 (1.00000)	-1.97254 (0.00200)	-1.22665 (0.10600)	-1.54169 (0.03300)	-1.69378 (0.02000)	-0.61631 (0.30300)
Fu`s Fs (*p*-value)	-25.40396 (0.00000)	0.00000 (0.22800)	-25.18616 (0.00100)	-1.60993 (0.11000)	-25.66400 (0.00000)	-17.96060 (0.00000)	-24.21740 (0.00000)

Ns: number of samples; h: number of haplotypes; Hd: haplotype diversity; *k*: average number of differences; π: nucleotide diversity

**Table 3:** Genetic diversity indices among clades of *Bombus morio* (Main: main clade; TS: Teodoro Sampaio clade) and *B. pauloensis* (N: north clade; C: central clade; S: south clade) obtained from 1575 bp of the following concatenated mitochondrial regions: *Cytochrome C oxidase I* (*COI*), *Cytochrome B* (*CytB*), the large ribosomal RNA subunit (*16S*), and cluster 4 of tRNA (covering a region of *COII* and *ATPase 8* genes and tRNA^lys^ and tRNA^asp^)

	Clades		Hd	k	Kxy	F_st_
*B. morio*	Main	TS	0.96697	4.54772	15.89779	0.82429
*B. pauloensis*	C	S	0.84808	2.57684	11.20899	0.77780
	C	N	0.88222	2.9693	9.50606	0.76508
	S	N	0.89429	3.12104	14.0295	0.79014

Hd: haplotype diversity; k: average number of differences; Kxy: average distance

#### Nuclear microsatellite data

All microsatellite loci were polymorphic for both species (Table 4). In *B. morio*’s Main clade, allele numbers per locus ranged from seven to 25 (mean 15.125). Expected and observed heterozygosity levels ranged from 0.658 to 0.932 (mean 0.835) and 0.621 to 0.891 (mean 0.755), respectively. Four loci were not in HWE (BM1, BM13, BM18 and BM20) and presented homozygote excess. F_IS_ value (0.104) was significant.

**Table 4:** Characterization of the genetic variability of clades from microsatellite data

Species	Clade	Locus	Ns	Na	Ar	Ho	He	*p-val*	Hom	F_IS_
*Bombus morio*	Main	BM1	66	12	11.939	0.742	0.871	0.0216	yes	0.155
		BM3	66	14	13.908	0.773	0.860	0.3329	no	0.109
		BM4	66	14	13.847	0.788	0.813	0.4699	no	0.039
		BM11	64	20	20.000	0.891	0.929	0.1365	no	0.050
		BM13	66	7	6.970	0.621	0.739	0.0009	yes	0.167
		BM17	66	14	13.968	0.636	0.658	0.4432	no	0.041
		BM18	66	15	14.939	0.803	0.883	0.0101	yes	0.099
		BM20	65	25	24.907	0.785	0.932	0.0025	yes	0.166
		Mean	65.625	15.125	15.060	0.755	0.835	-	-	0.104
*Bombus pauloensis*	C	BA2	21	10	9.808	0.571	0.729	0.0324	yes	0.239
		BA9	21	10	9.901	0.810	0.839	0.2017	no	0.059
		BA11	20	12	12.000	0.850	0.825	0.5260	no	-0.005
		BA15	21	7	6.905	0.714	0.710	0.2039	no	0.018
		BA8	21	12	11.760	0.905	0.814	0.5813	no	-0.087
		BA4	21	10	9.901	0.810	0.855	0.1245	no	0.077
		BA7	21	12	11.808	0.762	0.870	0.2422	no	0.148
		BA17	21	16	15.711	0.857	0.910	0.0877	no	0.083
		Mean	20.875	11.125	10.974	0.785	0.819	-	-	0.066
	N	BA2	41	12	8.595	0.415	0.664	0.0039	yes	0.386
		BA9	41	13	11.024	0.634	0.866	0.0000	yes	0.279
		BA11	41	6	5.327	0.366	0.595	0.0005	yes	0.395
		BA15	41	12	9.731	0.561	0.752	0.0006	yes	0.265
		BA8	41	16	13.449	0.732	0.906	0.0035	yes	0.204
		BA4	41	16	11.932	0.707	0.857	0.3316	yes	0.186
		BA7	41	15	12.847	0.683	0.883	0.0056	yes	0.238
		BA17	41	20	14.094	0.878	0.874	0.0965	no	0.008
		Mean	41	13.5	10.875	0.622	0.799	-	-	0.234
	S	BA2	34	8	7.039	0.441	0.522	0.0378	no	0.169
		BA9	34	11	10.299	0.765	0.875	0.0929	no	0.140
		BA11	34	23	17.330	0.706	0.906	0.0000	yes	0.235
		BA15	33	11	9.395	0.636	0.809	0.0831	yes	0.228
		BA8	34	13	11.360	0.824	0.870	0.4091	no	0.068
		BA4	33	14	11.872	0.727	0.881	0.0052	yes	0.189
		BA7	34	16	13.590	0.882	0.903	0.2410	no	0.037
		BA17	34	15	12.761	0.765	0.882	0.1047	yes	0.148
		Mean	33.75	13.875	11.706	0.718	0.831	-	-	0.150

Main: main clade found in *Bombus morio* from mitochondrial data; C, S and N: central, south, and north clades obtained in *B. pauloensis* from mitochondrial data. Ns: Number of samples; Na: effective number of alleles; Ar: allelic richness; Ho: observed heterozygosity; He: expected heterozygosity; *p-val*: *p*-value used to determine whether markers deviated from Hardy-Weinberg equilibrium (<0.05); Hom: homozygote excess, according to MicroChecker; F_is: _inbreeding coefficient

For *B. pauloensis*, the allele number per locus ranged from six to 23 (mean 12.917). Expected and observed heterozygosity levels ranged from 0.638 to 0.889 (mean 0.816) and 0.476 to 0.833 (mean 0.708), respectively. Allele richness was high (11.569). Within the C clade all loci were in HWE; homozygote excess was observed only in BA2. In the N clade, only BA4 and BA17 were in HWE; homozygote excess was found in all loci, except BA17. For the S clade, loci BA7, BA8, BA9, BA15 and BA17 were in HWE; loci BA4, BA11, BA15, and BA17 presented homozygote excess. The observed heterozygosity was lower than the expected in N, followed by the S and C clades. Allele richness was highest in the N and S clades. F_st_ values across each pair of clades were very low (0.025 C vs. N; 0.017 C vs. S; 0.019 N vs. S), indicating historical gene flow and lack of structure within the species. F_IS_ values were significant for the three clades, but higher in N and S (0.234 and 0.150) than C (0.066).

According to the Structure and Structure Harvester analyses, the best delta K value found for *B. morio* was 2 (Fig. 1d; Additional file 7). Yet, because a setup of K = 1 had the highest likelihood value (Additional file 8), and given that the software calculates the rate of change in the log likelihood, it will not output results for K = 1. The F_st_ values between the two K`s found were low (0.024). For *B. pauloensis* the best delta K value (Additional files 9 and 10) suggests the occurrence of four distinct nuclear clusters, but F_st_ values among them are low (0.025−0.048; Fig. 1h).

### Geographic distribution modeling

Distribution models of both species had high AUC levels (>0.97). Maximum temperature of the warmest month (Bio 5), precipitation of the warmest quarter (Bio 18), and temperature seasonality (Bio 4) were the climatic variables that most contributed to distribution models of both species. Together, these climatic variables contributed for 77.5% and 80% for *B. morio* and *B. pauloensis* distributions, respectively. These common climatic factors strongly support the geographic distribution overlap between these species and are consistent with observations that higher temperatures associated with low precipitation seem to limit the distribution of these bees.

**Fig. 2:**
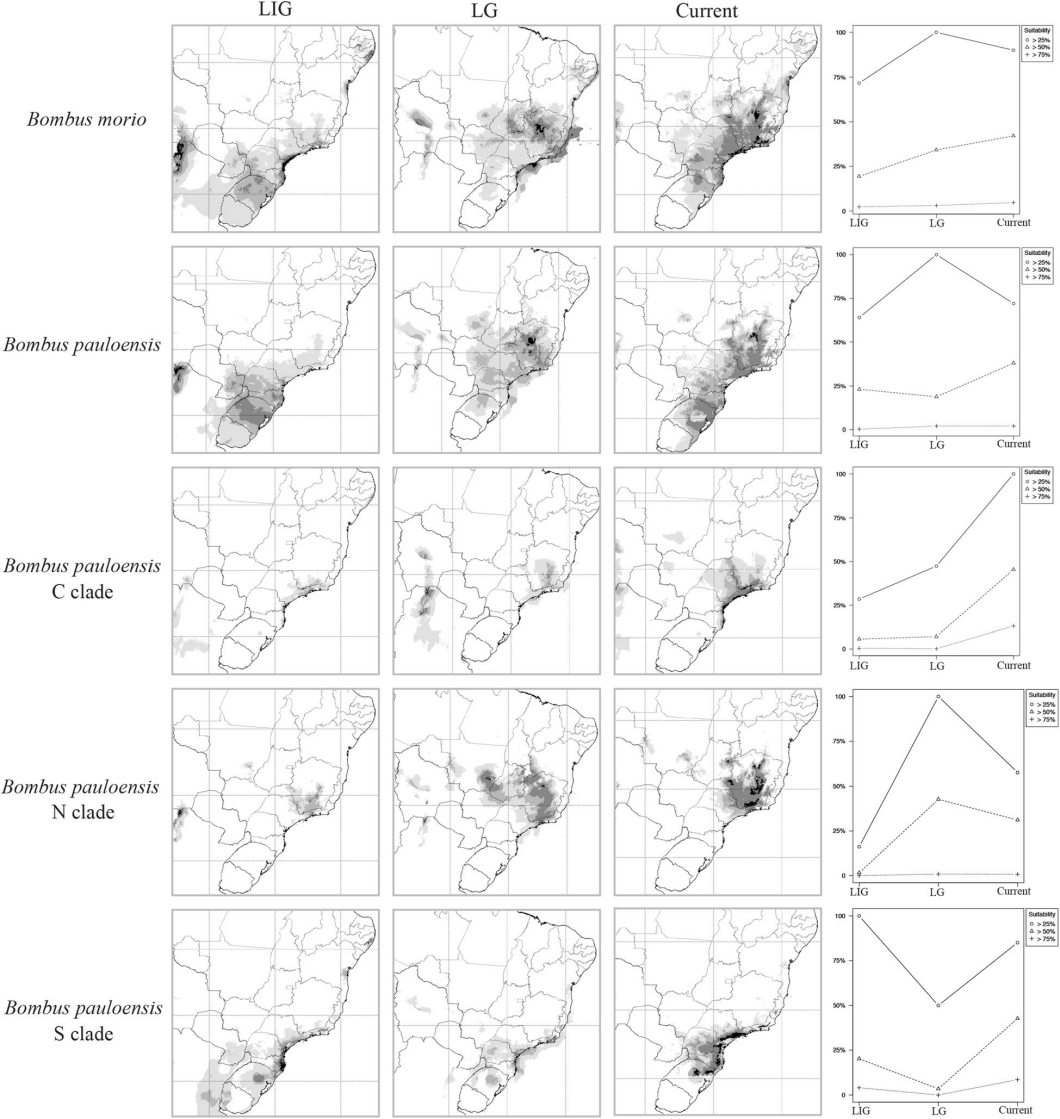
Geographic distribution modeling and area evaluation of *Bombus morio* and *B. pauloensis*, and the clades found in *B. pauloensis* from mitochondrial DNA data. C: central; N: north; S: south. LIG: Last Interglacial (~120,000–140,000 years BP); LGM: Last Glacial Maximum (~21,000 years BP). Graphics represent the distributional area reduction from LIG until current data, in suitability thresholds of maximum, medium and minimum probabilities (higher than 75%, higher than 50% and higher than 25%, respectively) of predicted presence. The axes of ordinates show the occupied area in a normalized ratio

For both species, the ENMs revealed population retraction in the LIG, followed by expansion in the LGM (Fig. 2). As an exploratory analysis, we performed distribution projections for each of the three clades found in *B. pauloensis* (C, N, and S) (Fig. 2). The clade C showed retraction to the south and southeast regions during the LIG and expansion to the north during the LGM. The clade N showed strong retraction in the distribution to the southeast region during the LIG and expansion to the north in the LGM. The clade S remained in the south during the LIG and the LGM (see Additional file 11 for climatic variables contributions for each species). We analyzed the suitability for each species and for the clades in *B. pauloensis* with three thresholds: maximum probability, corresponding to a predicted probability of the presence higher than 75%; medium probability, corresponding to probabilities higher than 50%; and minimum probability, representing probabilities over 25%. Based on this information, we evaluated the percentage reduction of species suitable area (Fig. 2).

### Environmental analysis

**Fig. 3:**
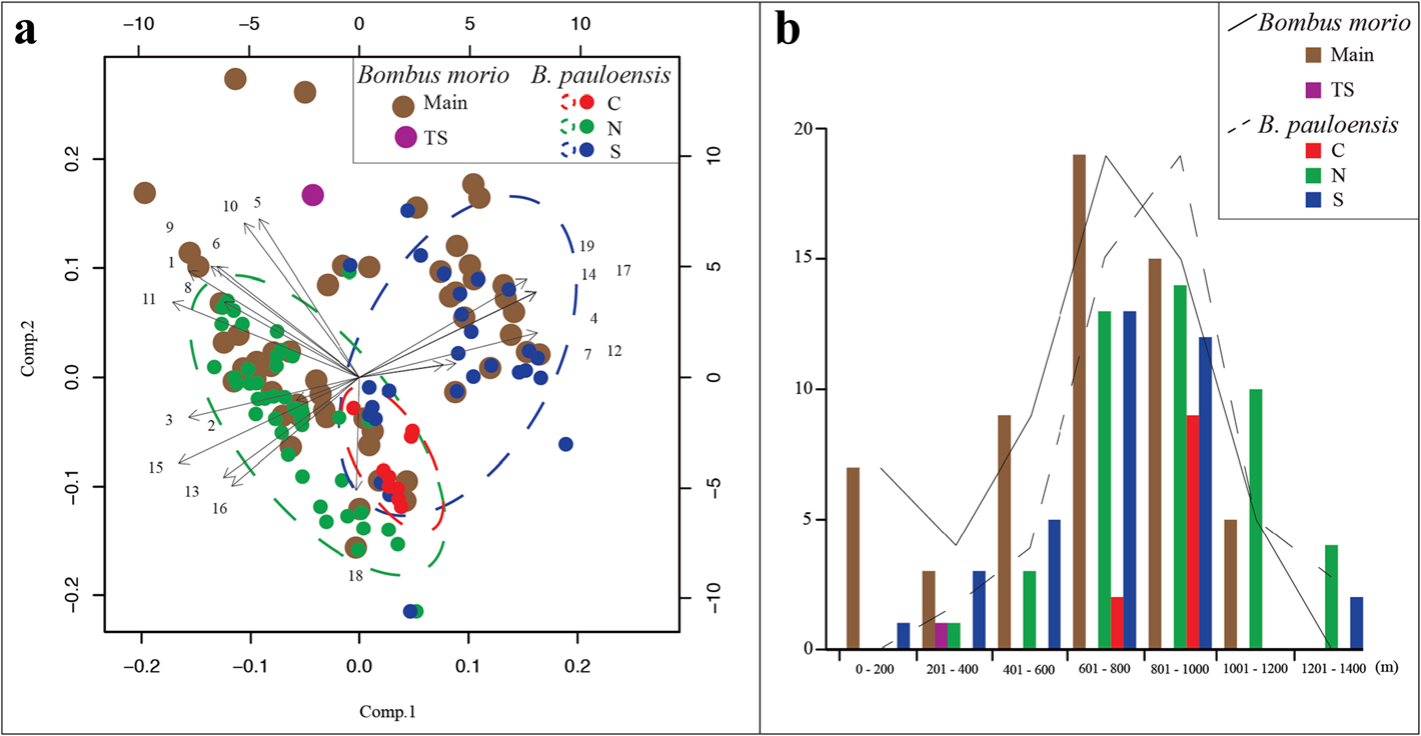
Environmental influence in the species distribution analysis*.* **a** PCA of the influence of bioclimatic variables (1−19, according to WorldClim) on the biogeographic patterns within *Bombus morio* and *B. pauloensis*. Comp. 1: temperature seasonality, precipitation of wettest month, precipitation of driest month, and precipitation of coldest quarter (4, 17, 14 and 19, respectively); Comp. 2: maximum temperature of warmest month, mean temperature of warmest quarter, minimum temperature of coldest month, and mean temperature of driest quarter (5, 10, 6 and 9, respectively); **b** number of observations in relation to altitude in *Bombus morio*, *B. pauloensis*, and their internal clades. Lines represent the tendency of observations sum by species and are not consistent with the scale

The distribution of these two species of bees is largely defined by climate. Principal components 1 and 2 explain 49.06 and 21.08% of the environmental variation of both species, totaling 70.14%. Bioclimatic variables most associated with the first principal component were temperature seasonality, precipitation of the wettest month, precipitation of the driest month, and precipitation of the coldest quarter (loading values of 0.286, 0.284, 0.283, and 0.269 respectively). For the second component, those bioclimatic variables most associated with the distribution of the species were maximum temperature of the warmest month, mean temperature of the warmest quarter, minimum temperature of the coldest month, and mean temperature of the driest quarter (loading values of 0.388, 0.378, 0.272 and 0.271 respectively; Fig. 3a).

We found no association between the range of *B. morio*’s main clade and the distribution points of the principal component analysis. According to the PCA and the species distribution, *B. morio* is associated with conditions represented by both sides of the components, occurring equally in the humid subtropical, high altitude tropical, and tropical savannah climates (Cfa, Cwa or Cwb and Aw, respectively, according to Köeppen-Geiger classifications). The Teodoro Sampaio region, however, is associated with the negative side of component 1 (mostly related to precipitation extremes) and with the positive side of component 2 (mostly related to temperature extremes). In a very distinct region, these samples are located in a transitional zone between Atlantic forest and Brazilian savanna. The extreme temperature of the warmer months is the bioclimatic variable that best differentiates this region.

*Bombus pauloensis* showed no association with the two principal components when analyzed as a whole. Nevertheless, the mitochondrial clades C, N, and S, with different frequencies of color patterns, occupy different geographical and climatic regions. The C clade is associated with the positive side of component 1 and the negative side of component 2. It occurs in high altitude tropical areas (above 500 m), mainly in the Serra do Mar region, characterized by mild temperatures (18−26 °C) during the entire year and high humidity. In summer, the maximum temperature rarely exceeds 30 °C. The N clade is associated with the negative sides of components 1 and 2 and occurs in tropical savanna climate. In this climate, the average temperature in all months of the year is over 18 °C; it has a very well defined dry and wet season. This clade occupies a warm region with extremes of humidity, therefore the first and second components from PCA does not influence its distribution. The S clade is associated with the positive sides of components 1 and 2, and occurs in humid subtropical climate. This type of climate is characterized by abundant rainfall in all months of the year (which justifies the positive side of component 1), but mostly in summer. The temperatures of the hottest month are above 22 °C, the temperatures in the coldest month are lower than 18 °C, and the average of the minimum temperature in winter is -3 °C, which explains the influence of temperature extremes in this clade.

Although both species have a preference for 601–1,000 m elevation regions, *B. morio* also occupies lower altitudes (0–200 m) while *B. pauloensis* seems to be more tolerant of higher elevation conditions (1,201–1,400 m). The C, N, and S clades were not correlated with any specific altitude value (Fig. 3b).

## Discussion

### Topological incongruence due to differences in dispersion

Topological congruence occurs when similar phylogeographic patterns are detected between species that responded in synchrony to the same historical events [67]. Mitochondrial data fail to support topological congruence between our two study species, and our findings are congruent with a hypothesis that the higher dispersion capacity of *B. morio* is responsible for the discord. Except for two samples collected in Teodoro Sampaio, which are deeply divergent from the species’ main clade and may represent a previously undescribed species (to be confirmed), *B. morio* shows evidence of ample gene flow, with no lineage structure in geographic or environmental space. Unlike patterns of structure detected in vertebrates of the Atlantic forest [24–30], a few mitochondrial haplotypes were as widely distributed as 2,000 km of distance. Lack of genetic differentiation, as found in *B. morio*, may be indicative of large population size, high dispersal capacity, or both. It is known that bumblebees can have large dispersal capacity, especially males [68–74]. In the case of *B. morio*, Moure & Sakagami [20] reported flights of over 2,500 m. Moreover, these bees are the most robust compared to other Brazilian species [20]. These results suggest *B. morio* as a large, panmictic species – or at least reflect historical panmixia and large population sizes. Despite rarely, panmixia has been observed in a few species, including bees [75, 76].

In contrast to the patterns observed in the high dispersal *B. morio*, we find high levels of mitochondrial lineage structure within *B. pauloensis.* This suggests that this later may present lower dispersal capability leading to geographically structured clades (C, N, and S) and lower overall levels of gene flow throughout its range. Frequencies of color patterns along the distribution of *B. pauloensis* differ among the mitochondrial clades found (completely black in N, with regular yellow stripes in S, and with both and intermediates morphotypes in C). A very similar morphological pattern was found in snakes of the *Bothrops jararaca* complex, in which intermediate morphologies were found in the state of São Paulo and may represent a hybrid zone between the southern and northern populations [30].

Microsatellite data showed no structure for either species. For *B. morio*, as the F_st_ values between the two K`s were low (0.024), we failed to detect population structure; microsatellite data hence corroborated the signal emerging from the mtDNA (Fig. 1d). In *B. pauloensis*, however, also no structure was verified: low nuclear F_st_ values were found between the four K`s found (0.025−0.048; Fig. 1h), in contrast with the mtDNA results. Low amounts of geographic structure in the microsatellite data seem to be common in widely ranged bees, as little or none have been reported, for instance, in *Amegilla dawsoni* [76], *Andrena vaga* [77], *Euglossa cordata* [78], *Osmia rufa* [79], and other *Bombus* species [80, 81, 82]. Such lack of structure has been attributed to extended male flight distance, except in cases of conspicuous barriers such as oceanic islands [83, 84], high mountains [85], or for species that had been isolated for a long period [86].

### Heteroplasmy

Although heteroplasmy is often suggested to be more common than thought [87], published data are scarce. Our tests indicate that heteroplasmy was present but did not influence the topology of *B. morio*. The same was reported in the phylogeographic study of the shrimp *Crangon crangon* [88], where original and phased datasets with heteroplasmy showed the same genetic structuration, probably because, as found here, the phased haplotypes within most individuals tended to be very closely related.

Heteroplasmy is difficult to address because multiple haplotypes presumably remain functional and lack any telltale signs in the sequence, such as stop codons or frame-shift mutations [89]. In bees, the presence of heteroplasmy has been flagged in *Apis dorsata* (Cao et al. [90] say “double peaks”), *Centris analis* [91], *Andrena tarsata*, *Colletes succinctus*, *Halictus rubicundis*, *H. tumulorum*, *Osmia aurulenta*, and *Sphecodes geoffrellus* [92]. However, its effect in bees has not been discussed extensively (but see Magnacca & Brown [89], which report heteroplasmy in the mtDNA of 21 of the 49 species of Hawaiian *Hylaeus*, at levels of 1−6% or more, with strong implications for the reliability of species identification through DNA barcodes).

### Bottleneck in LIG and expansion in LGM

Molecular data and species distribution models of these two bee species are consistent with demographic responses to environmental changes in the Late Quaternary. Paleodistribution modeling suggests contraction in the range of both species during the LIG, towards the presently colder southern and southeastern Brazil, followed by range expansion during the LGM. All analyses of genetic diversity indicate population bottleneck followed by rapid population growth and accumulation of mutations in both species: high numbers of haplotypes and haplotype diversity combined with few mutational points among them, negative and significant values of Fu`s Fs and Tajima`s D statistics, large values of Hd combined with low values of π [93] in mitochondrial data, homozygote excess, significant values of F_IS_, a high number of alleles per locus, and allele richness in the nuclear microsatellite data. Major diversification events, in both species, are dated back to the late Pleistocene (100,000−200,000 ya).

With respect to *B. morio*, it is possible that warmer periods of the early Pleistocene led to the isolation and differentiation of the lineage presently seen in Teodoro Sampaio. Widely distributed species with a history of genetic isolation at deeper evolutionary timescales, such as this one, often encompass cryptic species or geographically distinct lineages that possess unique adaptations or face different environmental pressures [94]. In fact, Teodoro Sampaio is located in an ecotone (a transitional zone between Atlantic forest and Brazilian savanna) that is isolated from the main clade distribution by low-lying areas. Ecotones are important for biodiversity since adaptive variation across environments is common in these areas [95]. However, differently from the mtDNA data, the single Teodoro Sampaio sample genotyped for microsatellite variation shares alleles with individuals whose mitochondrial lineages belong to the Main clade. This is not surprising given the stochasticity of the coalescent process, or may suggest that nuclear gene flow may have existed more recently, through males. More detailed studies of Teodoro Sampaio samples are needed to elucidate this question.

In *B. pauloensis*, levels of geographic structure (as shown by F_st_) and preliminary dating of the mitochondrial genealogy is congruent with a Late Pleistocene origin of the three major clades (C, N and S). Distribution models conducted for the species as a whole and separately for each clade corroborate a hypothesis of southern expansion during the LIG and a northern expansion in the LGM. Negative values of Fu`s Fs and Tajima`s D in the mitochondrial data statistics support this hypothesis of population expansion in the N and S clades. Moreover, while the C clade is in HWE according to nuclear microsatellite data, there is homozygote excess in the microsatellites of individuals belonging to the N and S mitochondrial clades. Microsatellite allele richness also was higher in the latter clades, suggesting a marked bottleneck and recent expansion. Differently from localities in the southern and northern most range of the species, those in Eastern São Paulo appear as a suitable region for the species irrespective of the time period modeled.

### A new phylogeographic scenario in state of São Paulo

The eastern portion of the state of São Paulo seems to represent the center of genetic diversity for *B. pauloensis*. This is supported by the presence, in this region, of all three mitochondrial lineages, all morphotypes, higher genetic diversity, the absence of homozygote excess in the microsatellite data, the HWE observed, the presence of transitional zones among different climates, and the higher levels of climatic stability over time.

Our analyses suggest that both *B. morio* and *B. pauloensis* contracted their ranges toward the southeast during the LIG, and underwent dramatic demographic expansion in the LGM. This contrast with the scenario suggested for lowland and mid-altitude Neotropical species, in which refuges during the LGM [24, 28, 29, 31, 32] and tectonic activity [25–27, 34] seem to be tied to the maintenance and generation of genetic diversity. Our study species reveal a different scenario for cold-associated species, whose distributions were likely larger during the LGM and which were isolated in “refuges” during the LIG, much like other cold-tolerant species of the Atlantic Forest [27, 32, 96, 97]. Bumblebees are cold adapted, and warm periods seemingly contributed to demographic contraction, while the opposite happened in periods of cooling.

### Current distribution

*Bombus morio* and *B. pauloensis* seem to have a preference for regions with higher elevations, which could be explained by their Nearctic origins of the South American *Bombus* species [39] and tolerance to cold climates. Given the absence of structure in the microsatellite data, no barriers to male gene flow are apparent. In contrast to the majority of bumblebee species, which have an annual life cycle (fertilized queens emerge from hibernation in late winter or early spring to found nests) [98, 99], *B. morio* and *B. pauloensis* do not hibernate. Year-round availability of food [22], tied to a biannual life cycle [100], may further increase gene flow.

For *B. pauloensis*, the mitochondrial clades C, N, and S are located in different climatic regions. The spatial distribution of mitochondrial and morphological diversity nonetheless suggest some level of gene flow across putative barriers structuring local diversity: individuals belonging to mitochondrial clades S and N can be found in the central São Paulo state, and higher morphological diversity is also found in this region. The corridor of higher altitude mountains found in eastern São Paulo may possibly provide a connection route among these three clades.

Parapatric diversification is the most suitable model to explain the distribution scenarios for the clades in *B. pauloensis*, isolated or semi-isolated from one another by different climatic conditions and altitude barriers. High-elevation habitat may also serve as an isolating mechanism in some *Bombus* species, as in *B. bifarius* [101], since populations at higher elevations are smaller and less well connected than those at lower elevations [102].

## Conclusions

Our results are congruent with a hypothesis of climatic oscillations during the Pleistocene having directly influenced the population demography of two widespread Neotropical bumblebees. Topologic congruence is not observed between their mitochondrial genealogies, as expected given the documented higher dispersal capacity of *B. morio. Bombus morio* is a very homogeneous species (morphological and genetically), showing no genetic structure in both mitochondrial and nuclear data, which suggest panmixia. Demographic expansions in the LGM, tied to its great dispersal ability, likely explain the high genetic diversity found in this species and the absence of genetic structure.

*Bombus pauloensis* also shows no structure in the microsatellite data, yet our sampling reveals three distinct mitochondrial lineages. Results of the demographic analyses and paleodistribution models are consistent with a scenario of southern expansion during the LIG and a northern expansion during the LGM. Eastern São Paulo have remained suitable for species occurrences during these distinct climatic periods, and today harbors most of the genetic and morphological diversity within the species range.

## Additional files

Additional file 1: Specimens with the four mitochondrial regions used (*Cytochrome C oxidase I*, *Cytochrome B*, the large ribosomal RNA subunit, and cluster 4 of tRNA) and collection data. Main: main clade; TS: Teodoro Sampaio clade; N: north clade; C: central clade; S: south clade. MS: specimens that had their microsatellites genotyped. (DOCX 92 kb)
Additional file 2: Molecular clock calibration phylogeny from sequences and calibration points (arrows) used by Hines [39]. Through this phylogeny, the dating for the clade *Thoracobombus* (highlighted), the common ancestral node that includes *Bombus morio* and *B. pauloensis*, was estimated at 13,5962 Ma. This dating was used to calibrate the phylogeny for each one of these species, once *B. pauloensis* was used as the outgroup of *B. morio* and vice versa. (DOCX 80 kb)
Additional file 3: Genotypes results for *Bombus morio*. (DOCX 29 kb)
Additional file 4: Genotypes results for *Bombus pauloensis.* (DOCX 35 kb)
Additional file 5: Georeferenced occurrence records for *Bombus morio* and *B. pauloensis* used for geographic distribution modeling. (DOCX 35 kb)
Additional file 6: Heteroplasmy found in the *Bombus morio* mitochondrial markers (undiscriminated nucleotides) characterized by double peaks, low phred quality scores, and synonymous divergence for the coding regions. (DOCX 981 kb)
Additional file 7: Delta K obtained for *Bombus morio* for each K. (DOCX 29 kb)
Additional file 8: Mean of likelihood obtained for *Bombus morio* for each K. (DOCX 37 kb)
Additional file 9: Delta K obtained for *Bombus pauloensis* for each K. (DOCX 30 kb)
Additional file 10: Mean of likelihood obtained for *Bombus pauloensis* for each K. (DOCX 37 kb)
Additional file 11: Contribution of each climatic variable in the geographic distribution of *Bombus morio* and *B. pauloensis*. (DOCX 15 kb)

## Abbreviations

*16S*: Large ribosomal RNA subunit
AUC: Area Under the Curve
C: central clade
*COI*: *Cytochrome C oxidase I*
*Cl4*: cluster 4 of tRNA (covering a region of COII and ATPase 8 genes and tRNAlys and tRNAasp)
*CytB*: *Cytochrome B*
F_IS_: Inbreeding coefficient
Hd: Haplotype diversity
He: Expected heterozygosity
Ho: Observed heterozygosity
HWE: Hardy–Weinberg equilibrium
k: Average number of nucleotide differences
K: Genetic groups
Kxy: Average distance
LGM: Last Glacial Maximum
LIG: Last Interglacial
Main: Main clade
MCMC: Markov chain Monte Carlo
N: North clade
NUMT: Nuclear mitochondrial DNA
PCA: Principal component analysis
PCRs: Polymerase chain reactions
Q: Proportion of genetic mixing of each individual
S: South clade
TS: Teodoro Sampaio clade
π: Nucleotide diversity
