# Supplementary material for: Comparative phylogeography in the Atlantic forest and Brazilian savannas: pleistocene fluctuations and dispersal shape spatial patterns in two bumblebees
Source: BMC Evol Biol. 2016 Dec 7;16:267. doi: 10.1186/s12862-016-0803-0 (PMC5142330; doi:10.1186/s12862-016-0803-0)
Supplement: Additional file 1: — Specimens with the four mitochondrial regions used (Cytochrome C oxidase I, Cytochrome B, the large ribosomal RNA subunit, and cluster 4 of tRNA) and collection data. Main: main clade; TS: Teodoro Sampaio clade; N: north clade; C: central clade; S: south clade. MS: specimens that had their microsatellites genotyped. (DOCX 92 kb) [file 12862_2016_803_MOESM1_ESM.docx]

**Additional file 1** – Specimens with the four mitochondrial regions used (*Cytochrome c oxidase I*, *Cytochrome B*, the large ribosomal RNA subunit and cluster 4 of tRNA) and collection data. **Main**: Main clade; **TS**: Teodoro Sampaio clade; **N**: north clade; **C**: central clade; **S**: south clade. **YES:** specimens that had their microsatellites genotyped.

| **Species Id** | **Species name** | **Collect city** | **State** | **Collect year** | **Collector** | **Collection** | **Voucher** | **Tissue conservation** | **LONG** | **LAT** | **Clade** | **Microsat.** |
| --- | --- | --- | --- | --- | --- | --- | --- | --- | --- | --- | --- | --- |
| 1 | *B. morio* | Ribeirão Preto | SP | 2009 | Aassis | LGEA | EF1 | frozen | -47.8211 | -21.1766 | Main |  |
| 2 | *B. morio* | Ribeirão Preto | SP | 2009 | Aassis | LGEA | EF2 | frozen | -47.8211 | -21.1766 | Main | yes |
| 10 | *B. morio* | Teresópolis | RJ | 2009 | FOFrancisco | LGEA | FOF226 | frozen | -42.9664 | -22.4123 | Main | yes |
| 11 | *B. morio* | Angra dos Reis | RJ | 2009 | FOFrancisco | LGEA | FOF211 | frozen | -44.3184 | -23.0101 | Main | yes |
| 13 | *B. morio* | Santa Lúcia | PR | 2009 | FOFrancisco | LGEA | FOF234 | frozen | -53.5706 | -25.4110 | Main | yes |
| 16 | *B. morio* | Santa Tereza do Oeste | PR | 2009 | FOFrancisco | LGEA | FOF216 | frozen | -53.6276 | -25.0527 | Main | yes |
| 18 | *B. morio* | São Paulo | SP | 2008 | EFrançoso | LGEA | EF18 | frozen | -46.6046 | -23.6052 | Main |  |
| 22 | *B. morio* | Teresópolis | RJ | 2009 | FOFrancisco | LGEA | FOF254 | frozen | -42.9664 | -22.4123 | Main | yes |
| 23 | *B. morio* | Angra dos Reis | RJ | 2009 | FOFrancisco | LGEA | FOF230 | frozen | -44.3184 | -23.0101 | Main | yes |
| 24 | *B. morio* | Petrópolis | RJ | 2009 | FOFrancisco | LGEA | FOF256 | frozen | -43.1826 | -22.5048 | Main | yes |
| 25 | *B. morio* | Céu Azul | PR | 2009 | FOFrancisco | LGEA | FOF219 | frozen | -53.8456 | -25.1498 | Main | yes |
| 26 | *B. morio* | Petrópolis | RJ | 2009 | FOFrancisco | LGEA | FOF229 | frozen | -43.1826 | -22.5048 | Main |  |
| 27 | *B. morio* | Santa Maria | PR | 2009 | FOFrancisco | LGEA | FOF253 | frozen | -52.9167 | -26.3333 | Main |  |
| 28 | *B. morio* | Apiaí | SP | 2009 | FOFrancisco | LGEA | FOF200 | frozen | -48.8432 | -24.5138 | Main | yes |
| 29 | *B. morio* | Ilha Bela | SP | 2009 | FOFrancisco | LGEA | FOF208 | frozen | -45.3239 | -23.9171 | Main | yes |
| 30 | *B. morio* | Teresópolis | RJ | 2009 | FOFrancisco | LGEA | FOF255 | frozen | -42.9664 | -22.4123 | Main |  |
| 31 | *B. morio* | Céu Azul | PR | 2009 | FOFrancisco | LGEA | FOF239 | frozen | -53.8456 | -25.1498 | Main | yes |
| 32 | *B. morio* | Ilha Bela | SP | 2009 | FOFrancisco | LGEA | FOF199 | frozen | -45.3239 | -23.9171 | Main | yes |
| 33 | *B. morio* | Angra dos Reis | RJ | 2009 | FOFrancisco | LGEA | FOF244 | frozen | -44.3184 | -23.0101 | Main | yes |
| 34 | *B. morio* | Petrópolis | RJ | 2009 | FOFrancisco | LGEA | FOF241 | frozen | -43.1826 | -22.5048 | Main |  |
| 35 | *B. morio* | Guaratuba | SP | 2009 | FOFrancisco | LGEA | FOF188 | frozen | -46.1285 | -23.8607 | Main |  |
| 36 | *B. morio* | Lindoeste | PR | 2009 | FOFrancisco | LGEA | FOF235 | frozen | -53.5761 | -25.2592 | Main | yes |
| 37 | *B. morio* | Santa Terezinha do Itaipu | PR | 2009 | FOFrancisco | LGEA | FOF224 | frozen | -54.4071 | -25.4489 | Main | yes |
| 39 | *B. morio* | Ibiúna | SP | 2010 | EFrançoso | LGEA | EF39 | frozen | -47.2257 | -23.6569 | Main |  |
| 40 | *B. morio* | Ibiúna | SP | 2010 | EFrançoso | LGEA | EF40 | frozen | -47.2257 | -23.6569 | Main | yes |
| 41 | *B. morio* | Ibiúna | SP | 2010 | EFrançoso | LGEA | EF41 | frozen | -47.2257 | -23.6569 | Main |  |
| 42 | *B. morio* | Santo Antonio do Barreiro | SP | 2010 | Hserafim | LGEA | EF42 | frozen | -44.5753 | -22.6454 | Main | yes |
| 60 | *B. morio* | Brasilândia de Minas | MG | 1996 | JDSouza | UFMG | 0431-1035 | pinned | -46.0152 | -17.0194 | Main |  |
| 71 | *B. morio* | Bocaiúva | MG | 1998 | FASilveira | UFMG | 2810-8247 | pinned | -43.8212 | -17.1152 | Main |  |
| 72 | *B. morio* | Curvelo | MG | 1998 | FASilveira | UFMG | 2823-8287 | pinned | -44.4468 | -18.7492 | Main | yes |
| 76 | *B. morio* | Chapada Gaúcha | MG | 2003 | AAAzevedo | UFMG | 8325-25415 | pinned | -45.4183 | -15.4684 | Main |  |
| 83 | *B. morio* | São Gonçalo do Rio Abaixo | MG | 2003 | FASilveira | UFMG | 9827-28980 | pinned | -43.3820 | -19.8281 | Main |  |
| 84 | *B. morio* | Grão Mogol | MG | 2005 | AAAzevedo | UFMG | 10239-29936 | pinned | -42.8939 | -16.5575 | Main | yes |
| 85 | *B. morio* | São Gonçalo do Rio Preto | MG | 2006 | AAAzevedo | UFMG | 10706-31475 | pinned | -43.3953 | -18.0066 | Main | yes |
| 86 | *B. morio* | Itacambira | MG | 2006 | Azevedo & Goulart | UFMG | 10731-31556 | pinned | -43.3115 | -17.0640 | Main | yes |
| 87 | *B. morio* | Joaquim Felício | MG | 2005 | FASilveira | UFMG | 10785-31818 | pinned | -44.1463 | -17.7797 | Main | yes |
| 88 | *B. morio* | Morretes | PR | 2001 | EABAlmeida | UFMG | 7743-23189 | pinned | -48.8316 | -25.4790 | Main | yes |
| 91 | *B. morio* | São Gonçalo do Rio Preto | MG | 2003 | LRRFaria Jr | UFMG | 10877-32109 | pinned | -43.3953 | -18.0066 | Main | yes |
| 92 | *B. morio* | Brasília | DF | 2006 | CFCardoso | UFMG | 11312-33667 | pinned | -47.9291 | -15.7802 | Main |  |
| 94 | *B. morio* | Itamonte | MG | 2006 | AAAzevedo | UFMG | 11215-33191 | pinned | -44.8680 | -22.2890 | Main |  |
| 95 | *B. morio* | Dores do Rio Preto | ES | 2006 | MFGoulart | UFMG | 11240-33287 | pinned | -41.8418 | -20.6932 | Main | yes |
| 96 | *B. morio* | Aiuruoca | MG | 2008 | RBMartines | UFMG | 13168-39038 | pinned | -44.6019 | -21.9294 | Main |  |
| 155 | *B. morio* | Jaboticatuvas | MG | 1989 | GMFaria | RPSP | RPSP-EF155 | pinned | -42.4167 | -18.7000 | Main |  |
| 171 | *B. morio* | São Paulo | SP | 2011 | LLLongo | LGEA | EF171 | frozen | -46.6046 | -23.6052 | Main |  |
| 172 | *B. morio* | Brotas | SP | 2011 | EFrançoso | LGEA | EF172 | frozen | -48.1223 | -22.2816 | Main | yes |
| 173 | *B. morio* | Brotas | SP | 2011 | EFrançoso | LGEA | EF173 | frozen | -48.1223 | -22.2816 | Main |  |
| 174 | *B. morio* | Brotas | SP | 2011 | EFrançoso | LGEA | EF174 | frozen | -48.1223 | -22.2816 | Main | yes |
| 177 | *B. morio* | Jaboticatuvas | MG | 2011 | EFrançoso | LGEA | EF177 | frozen | -42.4785 | -18.6893 | Main | yes |
| 180 | *B. morio* | Jaboticatuvas | MG | 2011 | EFrançoso | LGEA | EF180 | frozen | -42.3897 | -18.6893 | Main | yes |
| 182 | *B. morio* | Brasília | DF | 2009 | SCCappellari | UNB | 240508-12 | frozen | -47.8717 | -15.7614 | Main | yes |
| 183 | *B. morio* | Brasília | DF | 2008 | SCCappellari | UNB | 240708-13 | frozen | -47.8717 | -15.7614 | Main |  |
| 184 | *B. morio* | Brasília | DF | 2008 | SCCappellari | UNB | 240708-15 | frozen | -47.8717 | -15.7614 | Main | yes |
| 185 | *B. morio* | Brasília | DF | 2008 | SCCappellari | UNB | 240809 | frozen | -47.8717 | -15.7614 | Main |  |
| 187 | *B. morio* | Brasília | DF | 2009 | SCCappellari | UNB | 240809 | frozen | -47.8717 | -15.7614 | Main |  |
| 188 | *B. morio* | Brasília | DF | 2009 | SCCappellari | UNB | 240708-16 | frozen | -47.8717 | -15.7614 | Main |  |
| 189 | *B. morio* | Brasília | DF | 2008 | SCCappellari | UNB | 240708-23 | frozen | -47.8717 | -15.7614 | Main | yes |
| 190 | *B. morio* | Brasília | DF | 2008 | SCCappellari | UNB | 240708-14 | frozen | -47.8717 | -15.7614 | Main | yes |
| 191 | *B. morio* | Brasília | DF | 2008 | SCCappellari | UNB | 240708-12 | frozen | -47.8717 | -15.7614 | Main |  |
| 199 | *B. morio* | Jundiaí | SP | 2009 | PAPAntiqueira | Unesp | 260607-12 | frozen | -46.9728 | -23.2330 | Main |  |
| 200 | *B. morio* | Igrapiúna | BA | 2008 | EFrançoso | LGEA | 290309 | frozen | -39.1648 | -13.6818 | Main | yes |
| 201 | *B. morio* | Igrapiúna | BA | 2011 | EFrançoso | LGEA | 290309-2314 | frozen | -39.1648 | -13.6818 | Main | yes |
| 208 | *B. morio* | Igrapiúna | BA | 2011 | EFrançoso | LGEA | EF208 | frozen | -39.1648 | -13.6818 | Main | yes |
| 211 | *B. morio* | Igrapiúna | BA | 2011 | EFrançoso | LGEA | EF211 | frozen | -39.1648 | -13.6818 | Main |  |
| 217 | *B. morio* | Guarapari | ES | 2011 |  | UFES | (219a) - 58562 | frozen | -40.5049 | -20.6524 | Main | yes |
| 220 | *B. morio* | Londrina | PR | 2011 |  | LGEA | EF220 | frozen | -51.1737 | -23.2961 | Main | yes |
| 222 | *B. morio* | Londrina | PR | 2011 |  | LGEA | EF222 | frozen | -51.1737 | -23.2961 | Main | yes |
| 224 | *B. morio* | Londrina | PR | 2011 |  | LGEA | EF224 | frozen | -51.1737 | -23.2961 | Main | yes |
| 321 | *B. morio* | Itumirim | MG | 2007 | EFrançoso | LGEA | EF321 | frozen | -44.8704 | -21.3193 | Main | yes |
| 328 | *B. morio* | Uberlândia | MG | 2012 | EFrançoso | LGEA | EF328 | frozen | -48.2855 | -18.9440 | Main |  |
| 346 | *B. morio* | Brasília | DF | 2012 | EFrançoso | LGEA | EF346 | frozen | -47.8717 | -15.7614 | Main |  |
| 348 | *B. morio* | Brasília | DF | 2012 | EFrançoso | LGEA | EF348 | frozen | -47.8717 | -15.7614 | Main |  |
| 351 | *B. morio* | Brasília | DF | 2012 | EFrançoso | LGEA | EF351 | frozen | -47.8717 | -15.7614 | Main |  |
| 354 | *B. morio* | Goiás | GO | 2012 | EFrançoso | LGEA | EF354 | frozen | -50.1415 | -15.9331 | Main | yes |
| 355 | *B. morio* | Água limpa | SP | 2012 | EFrançoso | LGEA | EF355 | frozen | -48.3357 | -21.6932 | Main | yes |
| 356 | *B. morio* | Água limpa | SP | 2012 | EFrançoso | LGEA | EF356 | frozen | -48.3357 | -21.6932 | Main |  |
| 357 | *B. morio* | Água limpa | SP | 2012 | EFrançoso | LGEA | EF357 | frozen | -48.3357 | -21.6932 | Main |  |
| 358 | *B. morio* | Água limpa | SP | 2012 | EFrançoso | LGEA | EF358 | frozen | -48.3357 | -21.6932 | Main | yes |
| 359 | *B. morio* | Água limpa | SP | 2012 | EFrançoso | LGEA | EF359 | frozen | -48.3357 | -21.6932 | Main |  |
| 360 | *B. morio* | Água limpa | SP | 2012 | EFrançoso | LGEA | EF360 | frozen | -47.5459 | -22.3565 | Main |  |
| 362 | *B. morio* | Presidente Nereu | SC | 2013 | EFrançoso | LGEA | EF362 | frozen | -49.3296 | -27.2596 | Main | yes |
| 369 | *B. morio* | Presidente Nereu | SC | 2013 | EFrançoso | LGEA | EF369 | frozen | -49.3296 | -27.2596 | Main | yes |
| 391 | *B. morio* | Vacarias | RS | 2013 | EFrançoso | LGEA | EF391 | frozen | -51.0737 | -28.5439 | Main | yes |
| 393 | *B. morio* | Vacarias | RS | 2013 | EFrançoso | LGEA | EF393 | frozen | -51.0737 | -28.5439 | Main |  |
| 411 | *B. morio* | Caxias do Sul | RS | 2013 | EFrançoso | LGEA | EF411 | frozen | -51.2178 | -29.1344 | Main |  |
| 412 | *B. morio* | Caxias do Sul | RS | 2013 | EFrançoso | LGEA | EF412 | frozen | -51.2178 | -29.1344 | Main | yes |
| 413 | *B. morio* | Caxias do Sul | RS | 2013 | EFrançoso | LGEA | EF413 | frozen | -51.2178 | -29.1344 | Main |  |
| 414 | *B. morio* | Caxias do Sul | RS | 2013 | EFrançoso | LGEA | EF414 | frozen | -51.2178 | -29.1344 | Main |  |
| 415 | *B. morio* | Caxias do Sul | RS | 2013 | EFrançoso | LGEA | EF415 | frozen | -51.2178 | -29.1344 | Main |  |
| 416 | *B. morio* | Caxias do Sul | RS | 2013 | EFrançoso | LGEA | EF416 | frozen | -51.2178 | -29.1344 | Main |  |
| 417 | *B. morio* | Caxias do Sul | RS | 2013 | EFrançoso | LGEA | EF417 | frozen | -51.2178 | -29.1344 | Main |  |
| 418 | *B. morio* | Caxias do Sul | RS | 2013 | EFrançoso | LGEA | EF418 | frozen | -51.2172 | -29.1338 | Main |  |
| 419 | *B. morio* | Porto Alegre | RS | 2013 | EFrançoso | LGEA | EF419 | frozen | -51.1022 | -30.0291 | Main |  |
| 420 | *B. morio* | Porto Alegre | RS | 2013 | EFrançoso | LGEA | EF420 | frozen | -51.0979 | -30.1093 | Main |  |
| 421 | *B. morio* | Porto Alegre | RS | 2013 | EFrançoso | LGEA | EF421 | frozen | -51.1067 | -30.1043 | Main | yes |
| 422 | *B. morio* | Porto Alegre | RS | 2013 | EFrançoso | LGEA | EF422 | frozen | -51.1067 | -30.1043 | Main |  |
| 423 | *B. morio* | Porto Alegre | RS | 2013 | EFrançoso | LGEA | EF423 | frozen | -51.1067 | -30.1043 | Main | yes |
| 424 | *B. morio* | Porto Alegre | RS | 2013 | EFrançoso | LGEA | EF424 | frozen | -51.0904 | -30.1135 | Main |  |
| 425 | *B. morio* | Porto Alegre | RS | 2013 | EFrançoso | LGEA | EF425 | frozen | -51.0904 | -30.1135 | Main |  |
| 426 | *B. morio* | Porto Alegre | RS | 2013 | EFrançoso | LGEA | EF426 | frozen | -51.0904 | -30.1135 | Main |  |
| 427 | *B. morio* | Porto Alegre | RS | 2013 | EFrançoso | LGEA | EF427 | frozen | -51.0904 | -30.1135 | Main |  |
| 428 | *B. morio* | Lajeado | RS | 2013 | EFrançoso | LGEA | EF428 | frozen | -51.5802 | -29.2663 | Main |  |
| 429 | *B. morio* | Lajeado | RS | 2013 | EFrançoso | LGEA | EF429 | frozen | -51.5941 | -29.2644 | Main |  |
| 432 | *B. morio* | Lajeado | RS | 2013 | EFrançoso | LGEA | EF432 | frozen | -52.0318 | -29.2312 | Main |  |
| 433 | *B. morio* | Lajeado | RS | 2013 | EFrançoso | LGEA | EF433 | frozen | -52.0318 | -29.2312 | Main |  |
| 434 | *B. morio* | Lajeado | RS | 2013 | EFrançoso | LGEA | EF434 | frozen | -52.0318 | -29.2312 | Main |  |
| 435 | *B. morio* | Lajeado | RS | 2013 | EFrançoso | LGEA | EF435 | frozen | -52.0318 | -29.2312 | Main | yes |
| 436 | *B. morio* | Lajeado | RS | 2013 | EFrançoso | LGEA | EF436 | frozen | -52.0318 | -29.2312 | Main |  |
| 437 | *B. morio* | Lajeado | RS | 2013 | EFrançoso | LGEA | EF437 | frozen | -52.0318 | -29.2312 | Main |  |
| 438 | *B. morio* | Lajeado | RS | 2013 | EFrançoso | LGEA | EF438 | frozen | -52.0318 | -29.2312 | Main |  |
| 439 | *B. morio* | Lajeado | RS | 2013 | EFrançoso | LGEA | EF439 | frozen | -52.0318 | -29.2312 | Main |  |
| 440 | *B. morio* | Lajeado | RS | 2013 | EFrançoso | LGEA | EF440 | frozen | -52.0318 | -29.2312 | Main | yes |
| 441 | *B. morio* | Lajeado | RS | 2013 | EFrançoso | LGEA | EF441 | frozen | -52.0318 | -29.2312 | Main |  |
| 442 | *B. morio* | Lajeado | RS | 2013 | EFrançoso | LGEA | EF442 | frozen | -52.0318 | -29.2312 | Main | yes |
| 443 | *B. morio* | Lajeado | RS | 2013 | EFrançoso | LGEA | EF443 | frozen | -52.0318 | -29.2312 | Main |  |
| 444 | *B. morio* | Lajeado | RS | 2013 | EFrançoso | LGEA | EF444 | frozen | -52.0318 | -29.2312 | Main |  |
| 445 | *B. morio* | Lajeado | RS | 2013 | EFrançoso | LGEA | EF445 | frozen | -52.0318 | -29.2312 | Main |  |
| 447 | *B. morio* | Santo Antônio do Planalto | RS | 2013 | EFrançoso | LGEA | EF447 | frozen | -52.4148 | -28.2399 | Main | yes |
| 457 | *B. morio* | Chapecó | SC | 2013 | EFrançoso | LGEA | EF457 | frozen | -52.3685 | -27.0744 | Main |  |
| 458 | *B. morio* | Chapecó | SC | 2013 | EFrançoso | LGEA | EF458 | frozen | -52.3685 | -27.0744 | Main |  |
| 459 | *B. morio* | Chapecó | SC | 2013 | EFrançoso | LGEA | EF459 | frozen | -52.3685 | -27.0744 | Main | yes |
| 460 | *B. morio* | Chapecó | SC | 2013 | EFrançoso | LGEA | EF460 | frozen | -52.3685 | -27.0744 | Main | yes |
| 461 | *B. morio* | Chapecó | SC | 2013 | EFrançoso | LGEA | EF461 | frozen | -52.3685 | -27.0744 | Main | yes |
| 462 | *B. morio* | Chapecó | SC | 2013 | EFrançoso | LGEA | EF462 | frozen | -52.3685 | -27.0744 | Main | yes |
| 463 | *B. morio* | Chapecó | SC | 2013 | EFrançoso | LGEA | EF463 | frozen | -52.3685 | -27.0744 | Main | yes |
| 464 | *B. morio* | Chapecó | SC | 2013 | EFrançoso | LGEA | EF464 | frozen | -52.3685 | -27.0744 | Main | yes |
| 465 | *B. morio* | Chapecó | SC | 2013 | EFrançoso | LGEA | EF465 | frozen | -52.3685 | -27.0744 | Main |  |
| 466 | *B. morio* | Chapecó | SC | 2013 | EFrançoso | LGEA | EF466 | frozen | -52.3685 | -27.0744 | Main |  |
| 467 | *B. morio* | Chapecó | SC | 2013 | EFrançoso | LGEA | EF467 | frozen | -52.3685 | -27.0744 | Main |  |
| 468 | *B. morio* | Chapecó | SC | 2013 | EFrançoso | LGEA | EF468 | frozen | -52.3685 | -27.0744 | Main |  |
| 469 | *B. morio* | Chapecó | SC | 2013 | EFrançoso | LGEA | EF469 | frozen | -52.3685 | -27.0744 | Main |  |
| 470 | *B. morio* | Chapecó | SC | 2013 | EFrançoso | LGEA | EF470 | frozen | -52.3685 | -27.0744 | Main |  |
| 472 | *B. morio* | Chapecó | SC | 2013 | EFrançoso | LGEA | EF472 | frozen | -52.3685 | -27.0744 | Main |  |
| 473 | *B. morio* | Chapecó | SC | 2013 | EFrançoso | LGEA | EF473 | frozen | -52.3685 | -27.0744 | Main |  |
| 477 | *B. morio* | Chapecó | SC | 2013 | EFrançoso | LGEA | EF477 | frozen | -52.3674 | -27.0723 | Main |  |
| 478 | *B. morio* | Chapecó | SC | 2013 | EFrançoso | LGEA | EF478 | frozen | -52.3674 | -27.0723 | Main |  |
| 479 | *B. morio* | Chapecó | SC | 2013 | EFrançoso | LGEA | EF479 | frozen | -52.3674 | -27.0723 | Main |  |
| 480 | *B. morio* | Chapecó | SC | 2013 | EFrançoso | LGEA | EF480 | frozen | -52.3674 | -27.0723 | Main |  |
| 481 | *B. morio* | Chapecó | SC | 2013 | EFrançoso | LGEA | EF481 | frozen | -52.3674 | -27.0723 | Main |  |
| 482 | *B. morio* | Chapecó | SC | 2013 | EFrançoso | LGEA | EF482 | frozen | -52.3674 | -27.0723 | Main |  |
| 483 | *B. morio* | Chapecó | SC | 2013 | EFrançoso | LGEA | EF483 | frozen | -52.3674 | -27.0723 | Main |  |
| 484 | *B. morio* | Chapecó | SC | 2013 | EFrançoso | LGEA | EF484 | frozen | -52.3674 | -27.0723 | Main |  |
| 486 | *B. morio* | São Mateus do Sul | PR | 2013 | EFrançoso | LGEA | EF486 | frozen | -50.4223 | -26.0252 | Main | yes |
| 493 | *B. morio* | Ibiúna | SP | 2013 | EFrançoso | LGEA | EF493 | frozen | -47.2257 | -23.6569 | Main |  |
| 494 | *B. morio* | Ibiúna | SP | 2013 | EFrançoso | LGEA | EF494 | frozen | -47.2257 | -23.6569 | Main |  |
| 495 | *B. morio* | Ibiúna | SP | 2013 | EFrançoso | LGEA | EF495 | frozen | -47.2257 | -23.6569 | Main |  |
| 496 | *B. morio* | Ibiúna | SP | 2013 | EFrançoso | LGEA | EF496 | frozen | -47.2257 | -23.6569 | Main | yes |
| 497 | *B. morio* | Ibiúna | SP | 2013 | EFrançoso | LGEA | EF497 | frozen | -47.2257 | -23.6569 | Main |  |
| 498 | *B. morio* | Ibiúna | SP | 2013 | EFrançoso | LGEA | EF498 | frozen | -47.2257 | -23.6569 | Main | yes |
| 499 | *B. morio* | Ibiúna | SP | 2013 | EFrançoso | LGEA | EF499 | frozen | -47.2257 | -23.6569 | Main |  |
| 500 | *B. morio* | São Paulo | SP | 2013 | EFrançoso | LGEA | EF500 | frozen | -46.7301 | -23.5694 | Main |  |
| USP 1 | *B. morio* | São Paulo | SP | 2013 | EFrançoso | LGEA | EFUSP 1 | frozen | -46.7312 | -23.5658 | Main |  |
| USP 13 | *B. morio* | São Paulo | SP | 2009 | EFrançoso | LGEA | EFUSP 13 | frozen | -46.7312 | -23.5658 | Main |  |
| USP 14 | *B. morio* | São Paulo | SP | 2009 | EFrançoso | LGEA | EFUSP 14 | frozen | -46.7312 | -23.5658 | Main |  |
| USP 15 | *B. morio* | São Paulo | SP | 2009 | EFrançoso | LGEA | EFUSP 15 | frozen | -46.7312 | -23.5658 | Main |  |
| USP 16 | *B. morio* | São Paulo | SP | 2009 | EFrançoso | LGEA | EFUSP 16 | frozen | -46.7312 | -23.5658 | Main |  |
| USP 17 | *B. morio* | São Paulo | SP | 2010 | EFrançoso | LGEA | EFUSP 17 | frozen | -46.7312 | -23.5658 | Main |  |
| USP 18 | *B. morio* | São Paulo | SP | 2010 | LRSantiago | LGEA | EFUSP 18 | frozen | -46.7312 | -23.5658 | Main | yes |
| USP 4 | *B. morio* | São Paulo | SP | 2009 | EFrançoso | LGEA | EFUSP 4 | frozen | -46.7312 | -23.5658 | Main |  |
| USP 6 | *B. morio* | São Paulo | SP | 2009 | EFrançoso | LGEA | EFUSP 6 | frozen | -46.7312 | -23.5658 | Main |  |
| USP 7 | *B. morio* | São Paulo | SP | 2009 | EFrançoso | LGEA | EFUSP 7 | frozen | -46.7312 | -23.5658 | Main |  |
| USP 8 | *B. morio* | São Paulo | SP | 2009 | EFrançoso | LGEA | EFUSP 8 | frozen | -46.7312 | -23.5658 | Main |  |
| USP 9 | *B. morio* | São Paulo | SP | 2009 | EFrançoso | LGEA | EFUSP 9 | frozen | -46.7312 | -23.5658 | Main |  |
| USP19 | *B. morio* | São Paulo | SP | 2010 | EFrançoso | LGEA | EFUSP19 | frozen | -46.7312 | -23.5658 | Main |  |
| USP20 | *B. morio* | São Paulo | SP | 2011 | EFrançoso | LGEA | EFUSP20 | frozen | -46.7312 | -23.5658 | Main |  |
| USP21 | *B. morio* | São Paulo | SP | 2011 | EFrançoso | LGEA | EFUSP21 | frozen | -46.7312 | -23.5658 | Main |  |
| USP22 | *B. morio* | São Paulo | SP | 2011 | LRSantiago | LGEA | EFUSP22 | frozen | -46.7312 | -23.5658 | Main | yes |
| USP26 | *B. morio* | São Paulo | SP | 2011 | EFrançoso | LGEA | EFUSP26 | frozen | -46.7312 | -23.5658 | Main |  |
| USP30 | *B. morio* | São Paulo | SP | 2011 | EFrançoso | LGEA | EFUSP30 | frozen | -46.7312 | -23.5658 | Main |  |
| USP31 | *B. morio* | São Paulo | SP | 2011 | EFrançoso | LGEA | EFUSP31 | frozen | -46.7312 | -23.5658 | Main |  |
| USP32 | *B. morio* | São Paulo | SP | 2011 | EFrançoso | LGEA | EFUSP32 | frozen | -46.7312 | -23.5658 | Main |  |
| USP33 | *B. morio* | São Paulo | SP | 2011 | EFrançoso | LGEA | EFUSP33 | frozen | -46.7312 | -23.5658 | Main |  |
| USP35 | *B. morio* | São Paulo | SP | 2011 | WAugusto | LGEA | EFUSP35 | frozen | -46.7312 | -23.5658 | Main |  |
| USP36 | *B. morio* | São Paulo | SP | 2011 | WAugusto | LGEA | EFUSP36 | frozen | -46.7312 | -23.5658 | Main |  |
| USP38 | *B. morio* | São Paulo | SP | 2012 | WAugusto | LGEA | EFUSP38 | frozen | -46.7312 | -23.5658 | Main |  |
| USP39 | *B. morio* | São Paulo | SP | 2012 | WAugusto | LGEA | EFUSP39 | frozen | -46.7312 | -23.5658 | Main |  |
| USP41 | *B. morio* | São Paulo | SP | 2012 | WAugusto | LGEA | EFUSP41 | frozen | -46.7312 | -23.5658 | Main |  |
| USP42 | *B. morio* | São Paulo | SP | 2012 | WAugusto | LGEA | EFUSP42 | frozen | -46.7312 | -23.5658 | Main |  |
| USP44 | *B. morio* | São Paulo | SP | 2012 | WAugusto | LGEA | EFUSP44 | frozen | -46.7312 | -23.5658 | Main |  |
| USP46 | *B. morio* | São Paulo | SP | 2012 | WAugusto | LGEA | EFUSP46 | frozen | -46.7312 | -23.5658 | Main |  |
| USP47 | *B. morio* | São Paulo | SP | 2012 | WAugusto | LGEA | EFUSP47 | frozen | -46.7312 | -23.5658 | Main |  |
| USP48 | *B. morio* | São Paulo | SP | 2012 | WAugusto | LGEA | EFUSP48 | frozen | -46.7312 | -23.5658 | Main |  |
| 12 | *B. morio* | Teodoro Sampaio | SP | 2009 | FOFrancisco | LGEA | FOF223 | frozen | -52.1854 | -22.5320 | TS |  |
| 15 | *B. morio* | Teodoro Sampaio | SP | 2009 | FOFrancisco | LGEA | FOF249 | frozen | -52.1854 | -22.5320 | TS | yes |
| 170 | *B. pauloensis* | Guaratuba | SP | 2011 | FOFrancisco | LGEA | FOF170 | frozen | -47.4294 | -24.1264 | C | yes |
| 230 | *B. pauloensis* | Iguape | SP | 2011 | FOFrancisco | LGEA | FOF811 | frozen | -46.4952 | -23.3581 | C | yes |
| 231 | *B. pauloensis* | Iguape | SP | 2011 | FOFrancisco | LGEA | FOF819 | frozen | -46.4737 | -23.3877 | C | yes |
| 232 | *B. pauloensis* | Iguape | SP | 2011 | FOFrancisco | LGEA | FOF828 | frozen | -46.4999 | -23.3611 | C | yes |
| 233 | *B. pauloensis* | Iguape | SP | 2011 | FOFrancisco | LGEA | FOF830 | frozen | -46.4238 | -23.2799 | C | yes |
| 235 | *B. pauloensis* | Iguape | SP | 2011 | FOFrancisco | LGEA | FOF832 | frozen | -46.4238 | -23.2799 | C | yes |
| 236 | *B. pauloensis* | Ilha comprida | SP | 2011 | FOFrancisco | LGEA | FOF833 | frozen | -46.4604 | -23.2721 | C | yes |
| 237 | *B. pauloensis* | Iguape | SP | 2011 | FOFrancisco | LGEA | FOF835 | frozen | -46.4259 | -23.2865 | C | yes |
| 238 | *B. pauloensis* | Ilha comprida | SP | 2011 | FOFrancisco | LGEA | FOF836 | frozen | -46.4820 | -23.2850 | C | yes |
| 243 | *B. pauloensis* | Ilha comprida | SP | 2011 | FOFrancisco | LGEA | FOF848 | frozen | -46.4820 | -23.2850 | C | yes |
| 246 | *B. pauloensis* | Guaratuba | PR | 2011 | FOFrancisco | LGEA | FOF852 | frozen | -47.4217 | -24.0850 | C | yes |
| 248 | *B. pauloensis* | Guaratuba | PR | 2011 | FOFrancisco | LGEA | FOF858 | frozen | -47.4217 | -24.0850 | C | yes |
| 259 | *B. pauloensis* | Matinhos | PR | 2011 | FOFrancisco | LGEA | FOF1877 | frozen | -48.5784 | -22.8904 | C | yes |
| 266 | *B. pauloensis* | Iguape | SP | 2011 | FOFrancisco | LGEA | FOF1865 | frozen | -46.4999 | -23.3611 | C | yes |
| 268 | *B. pauloensis* | Iguape | SP | 2011 | FOFrancisco | LGEA | FOF1872 | frozen | -46.4999 | -23.3611 | C | yes |
| 270 | *B. pauloensis* | Matinhos | PR | 2011 | FOFrancisco | LGEA | FOF1876 | frozen | -48.5784 | -22.8904 | C | yes |
| 271 | *B. pauloensis* | Iguape | SP | 2011 | FOFrancisco | LGEA | FOF1880 | frozen | -46.4999 | -23.3611 | C | yes |
| 272 | *B. pauloensis* | Iguape | SP | 2011 | FOFrancisco | LGEA | FOF1881 | frozen | -46.4999 | -23.3611 | C | yes |
| 273 | *B. pauloensis* | Ilha Comprida | SP | 2011 | FOFrancisco | LGEA | FOF1882 | frozen | -46.4955 | -23.2790 | C | yes |
| 274 | *B. pauloensis* | Iguape | SP | 2011 | FOFrancisco | LGEA | FOF1886 | frozen | -46.4999 | -23.3611 | C | yes |
| 277 | *B. pauloensis* | Iguape | SP | 2011 | FOFrancisco | LGEA | FOF1917 | frozen | -46.4999 | -23.3611 | C | yes |
| 9 | *B. pauloensis* | Ribeirão Preto | SP | 2007 | Aassis | LGEA | EF9 | frozen | -47.8211 | -21.1766 | N | yes |
| 45 | *B. pauloensis* | Gonçalves | MG | 2006 | FASilveira | UFMG | 13439-39717 | pinned | -45.8544 | -22.6589 | N | yes |
| 47 | *B. pauloensis* | Olhos d`água | MG | 2006 | FASilveira | UFMG | 10908-32259 | pinned | -43.5754 | -17.3958 | N | yes |
| 48 | *B. pauloensis* | Camanducaia | MG | 2004 | FASilveira | UFMG | 10893-32199 | pinned | -46.1455 | -22.7553 | N |  |
| 50 | *B. pauloensis* | Baependi | MG | 2008 | RBMartines | UFMG | 13188-39081 | pinned | -44.8914 | -21.9596 | N |  |
| 51 | *B. pauloensis* | Aiuruoca | MG | 2008 | RBMartines | UFMG | 13175-39053 | pinned | -44.6019 | -21.9294 | N | yes |
| 52 | *B. pauloensis* | Aiuruoca | MG | 2008 | RBMartines | UFMG | 13173-39046 | pinned | -44.6019 | -21.9294 | N | yes |
| 53 | *B. pauloensis* | Alto Paraíso de Goiás | GO | 2006 | AAAzevedo | UFMG | 11277-33540 | pinned | -47.5215 | -14.1336 | N | yes |
| 54 | *B. pauloensis* | Alto Paraíso de Goiás | GO | 2006 | MFGoulart | UFMG | 11257-33383 | pinned | -47.5215 | -14.1336 | N |  |
| 55 | *B. pauloensis* | Alto Paraíso de Goiás | GO | 2006 | AAAzevedo | UFMG | 11241-33291 | pinned | -47.5215 | -14.1336 | N | yes |
| 56 | *B. pauloensis* | Dores do Rio Preto | ES | 2006 | MFGoulart | UFMG | 11233-33269 | pinned | -41.8418 | -20.6932 | N | yes |
| 57 | *B. pauloensis* | Dores do Rio Preto | ES | 2006 | MFGoulart | UFMG | 11233-33268 | pinned | -41.8418 | -20.6932 | N | yes |
| 58 | *B. pauloensis* | Alto Caparaó | MG | 2006 | AAAzevedo | UFMG | 11225-33247 | pinned | -41.8746 | -20.4331 | N | yes |
| 59 | *B. pauloensis* | Lima Duarte | MG | 2006 | AAAzevedo | UFMG | 11205-33142 | pinned | -43.8999 | -21.7396 | N |  |
| 62 | *B. pauloensis* | Itatiaia | RJ | 2007 | AAAzevedo | UFMG | 12803-37806 | pinned | -44.5608 | -22.4958 | N | yes |
| 63 | *B. pauloensis* | Itatiaia | RJ | 2007 | AAAzevedo | UFMG | 12803-37807 | pinned | -44.5608 | -22.4958 | N |  |
| 64 | *B. pauloensis* | Itatiaia | RJ | 2007 | MLoureiro | UFMG | 12806-37813 | pinned | -44.5608 | -22.4958 | N |  |
| 65 | *B. pauloensis* | São Roque de Minas | MG | 2007 | AAAzevedo | UFMG | 12944-3872 | pinned | -46.3671 | -20.2328 | N |  |
| 66 | *B. pauloensis* | Dores do Rio Preto | ES | 2007 | MFGoulart | UFMG | 12947-38292 | pinned | -41.8418 | -20.6932 | N | yes |
| 67 | *B. pauloensis* | Alto Paraíso de Goiás | GO | 2007 | MFGoulart | UFMG | 13001-38481 | pinned | -47.5215 | -14.1336 | N |  |
| 68 | *B. pauloensis* | Alto Paraíso de Goiás | GO | 2007 | MFGoulart | UFMG | 13017-38569 | pinned | -47.5215 | -14.1336 | N | yes |
| 80 | *B. pauloensis* | Santana do Riacho | MG | 2000 | EFMorato | UFMG | 7222-21464 | pinned | -43.6803 | -19.1170 | N |  |
| 93 | *B. pauloensis* | Ibirité | MG | 2004 | YAntonini | UFMG | 11403-33893 | pinned | -44.0565 | -20.0195 | N |  |
| 103 | *B. pauloensis* | Sabará | MG | 1996 | FASilveira | UFMG | 0676-1569 | pinned | -43.8048 | -19.8892 | N |  |
| 104 | *B. pauloensis* | Belo Horizonte | MG | 1996 | RMCarmo | UFMG | 0294-0844 | pinned | -43.9657 | -19.8157 | N |  |
| 105 | *B. pauloensis* | Belo Horizonte | MG | 1997 | JDamasceno | UFMG | 1106-2851 | pinned | -43.9657 | -19.8157 | N | yes |
| 106 | *B. pauloensis* | Santana do Riacho | MG | 1996 | CHVasconcelos | UFMG | 0687-1649 | pinned | -43.6803 | -19.1170 | N |  |
| 110 | *B. pauloensis* | itamarandiba | MG | 1998 | FASilveira | UFMG | 2035-6288 | pinned | -42.8610 | -17.8567 | N | yes |
| 111 | *B. pauloensis* | Caeté | MG | 1998 | FASilveira | UFMG | 2224-7011 | pinned | -43.6698 | -19.8806 | N | yes |
| 118 | *B. pauloensis* | Santa Bárbara | MG | 1999 | FASilveira | UFMG | 4921-13503 | pinned | -43.4142 | -19.9604 | N |  |
| 121 | *B. pauloensis* | Brasília | DF | 1999 | LMBreyer | UFMG | 4246-13459 | pinned | -47.9291 | -15.7802 | N |  |
| 122 | *B. pauloensis* | Bocaiúva | MG | 1999 | AAAzevedo | UFMG | 5794-15825 | pinned | -43.8212 | -17.1152 | N | yes |
| 124 | *B. pauloensis* | Nova Lima | MG | 1999 | MPompeu | UFMG | 4569-12785 | pinned | -43.8463 | -19.9876 | N | yes |
| 126 | *B. pauloensis* | Bocaiúva | MG | 2002 | AAAzevedo | UFMG | 8047-24510 | pinned | -43.8212 | -17.1152 | N | yes |
| 132 | *B. pauloensis* | Itacambira | MG | 2004 | AAAzevedo | UFMG | 10228-29907 | pinned | -43.3115 | -17.0640 | N | yes |
| 133 | *B. pauloensis* | Diamantina | MG | 2005 | Azevedo& Goulart | UFMG | 10594-31031 | pinned | -43.6110 | -18.2381 | N | yes |
| 134 | *B. pauloensis* | Francisco Dumont | MG | 2005 | Azevedo & Silveira | UFMG | 10634-31260 | pinned | -44.2422 | -17.2946 | N |  |
| 135 | *B. pauloensis* | Buenópolis | MG | 2005 | Azevedo & Goulart | UFMG | 10755-31684 | pinned | -44.1728 | -17.8698 | N | yes |
| 136 | *B. pauloensis* | Serranópolis de Minas | MG | 2006 | AAAzevedo | UFMG | 10784-31809 | pinned | -42.8696 | -15.8126 | N |  |
| 137 | *B. pauloensis* | Brasília | DF | 2006 | CLYarrita | UFMG | 11298-33639 | pinned | -47.9291 | -15.7802 | N |  |
| 178 | *B. pauloensis* | Jaboticatuvas | MG | 2011 | EFrançoso | LGEA | EF178 | frozen | -42.3897 | -18.6893 | N | yes |
| 179 | *B. pauloensis* | Jaboticatuvas | MG | 2011 | EFrançoso | LGEA | EF179 | frozen | -42.3897 | -18.6893 | N | yes |
| 181 | *B. pauloensis* | Brasília | DF | 2011 | SCCappellari | UNB | 24019-1 | frozen | -47.8717 | -15.7614 | N |  |
| 186 | *B. pauloensis* | Brasília | DF | 2009 | SCCappellari | UNB | 240809 | frozen | -47.8717 | -15.7614 | N | yes |
| 192 | *B. pauloensis* | Brasília | DF | 2008 | SCCappellari | UNB | 240809 | frozen | -47.8717 | -15.7614 | N |  |
| 193 | *B. pauloensis* | Brasília | DF | 2009 | SCCappellari | UNB | 240809 | frozen | -47.8717 | -15.7614 | N |  |
| 194 | *B. pauloensis* | Brasília | DF | 2009 | SCCappellari | UNB | 030809-2584 | frozen | -47.8717 | -15.7614 | N | yes |
| 195 | *B. pauloensis* | Brasília | DF | 2005 | SCCappellari | UNB | 100809-12 | frozen | -47.8717 | -15.7614 | N |  |
| 196 | *B. pauloensis* | Brasília | DF | 2005 | SCCappellari | UNB | 170809-2584 | frozen | -47.8717 | -15.7614 | N |  |
| 197 | *B. pauloensis* | Brasília | DF | 2009 | SCCappellari | UNB | 170809-2584 | frozen | -47.8717 | -15.7614 | N |  |
| 198 | *B. pauloensis* | Brasília | DF | 2009 | SCCappellari | UNB | 220309-2532 | frozen | -47.8717 | -15.7614 | N | yes |
| 223 | *B. pauloensis* | Londrina | PR | 2011 |  | LGEA | EF223 | frozen | -51.1737 | -23.2961 | N | yes |
| 226 | *B. pauloensis* | Itatiaia | RJ | 2010 | FOFrancisco | LGEA | FOF459 | frozen | -43.4381 | -21.5081 | N | yes |
| 227 | *B. pauloensis* | Itatiaia | RJ | 2010 | FOFrancisco | LGEA | FOF464 | frozen | -43.4408 | -21.5142 | N |  |
| 228 | *B. pauloensis* | Itatiaia | RJ | 2010 | FOFrancisco | LGEA | FOF465 | frozen | -43.4381 | -21.5081 | N |  |
| 250 | *B. pauloensis* | Resende | RJ | 2011 | FOFrancisco | LGEA | FOF1518 | frozen | -43.4647 | -21.5727 | N | yes |
| 251 | *B. pauloensis* | Itatiaia | RJ | 2011 | FOFrancisco | LGEA | FOF1525 | frozen | -43.4347 | -21.5008 | N |  |
| 252 | *B. pauloensis* | Itanhandu | MG | 2011 | FOFrancisco | LGEA | FOF1565 | frozen | -43.0598 | -21.7004 | N | yes |
| 253 | *B. pauloensis* | Passa Quatro | MG | 2011 | FOFrancisco | LGEA | FOF1843 | frozen | -44.9692 | -22.3846 | N | yes |
| 254 | *B. pauloensis* | Itamonte | MG | 2011 | FOFrancisco | LGEA | FOF1851 | frozen | -44.8680 | -22.2890 | N | yes |
| 255 | *B. pauloensis* | Resende | RJ | 2011 | FOFrancisco | LGEA | FOF1852 | frozen | -44.4567 | -22.4635 | N |  |
| 256 | *B. pauloensis* | Resende | RJ | 2011 | FOFrancisco | LGEA | FOF1853 | frozen | -44.4567 | -22.4635 | N |  |
| 257 | *B. pauloensis* | Resende | RJ | 2011 | FOFrancisco | LGEA | FOF1854 | frozen | -44.4567 | -22.4635 | N |  |
| 258 | *B. pauloensis* | Itatiaia | RJ | 2011 | FOFrancisco | LGEA | FOF1855 | frozen | -44.5608 | -22.4958 | N |  |
| 260 | *B. pauloensis* | Itamonte | SP | 2011 | FOFrancisco | LGEA | FOF1858 | frozen | -44.8680 | -22.2890 | N |  |
| 261 | *B. pauloensis* | Itamonte | SP | 2011 | FOFrancisco | LGEA | FOF1859 | frozen | -44.8680 | -22.2890 | N |  |
| 262 | *B. pauloensis* | Itamonte | SP | 2011 | FOFrancisco | LGEA | FOF1860 | frozen | -44.8680 | -22.2890 | N |  |
| 263 | *B. pauloensis* | Resende | RJ | 2011 | FOFrancisco | LGEA | FOF1861 | frozen | -44.4567 | -22.4635 | N | yes |
| 264 | *B. pauloensis* | Itatiaia | RJ | 2011 | FOFrancisco | LGEA | FOF1862 | frozen | -44.5608 | -22.4958 | N |  |
| 265 | *B. pauloensis* | Itatiaia | RJ | 2011 | FOFrancisco | LGEA | FOF1863 | frozen | -44.5608 | -22.4958 | N |  |
| 267 | *B. pauloensis* | Resende | RJ | 2011 | FOFrancisco | LGEA | FOF1867 | frozen | -44.4567 | -22.4635 | N |  |
| 275 | *B. pauloensis* | Itamonte | MG | 2011 | FOFrancisco | LGEA | FOF1887 | frozen | -44.8680 | -22.2890 | N |  |
| 322 | *B. pauloensis* | Carrancas | MG | 2012 | EFrançoso | LGEA | EF322 | frozen | -44.6402 | -21.4821 | N | yes |
| 323 | *B. pauloensis* | Uberlândia | MG | 2012 | EFrançoso | LGEA | EF323 | frozen | -48.2547 | -18.8830 | N | yes |
| 324 | *B. pauloensis* | Uberlândia | MG | 2012 | EFrançoso | LGEA | EF324 | frozen | -48.2998 | -18.9234 | N | yes |
| 325 | *B. pauloensis* | Uberlândia | MG | 2012 | EFrançoso | LGEA | EF325 | frozen | -48.2998 | -18.9234 | N | yes |
| 326 | *B. pauloensis* | Uberlândia | MG | 2012 | EFrançoso | LGEA | EF326 | frozen | -48.3016 | -18.9209 | N |  |
| 327 | *B. pauloensis* | Uberlândia | MG | 2012 | EFrançoso | LGEA | EF327 | frozen | -48.2855 | -18.9440 | N |  |
| 329 | *B. pauloensis* | Uberlândia | MG | 2012 | EFrançoso | LGEA | EF329 | frozen | -48.2855 | -18.9440 | N |  |
| 330 | *B. pauloensis* | Uberlândia | MG | 2012 | EFrançoso | LGEA | EF330 | frozen | -48.2855 | -18.9440 | N |  |
| 331 | *B. pauloensis* | Uberlândia | MG | 2012 | EFrançoso | LGEA | EF331 | frozen | -48.2855 | -18.9440 | N |  |
| 332 | *B. pauloensis* | Brasília | DF | 2012 | EFrançoso | LGEA | EF332 | frozen | -47.9804 | -15.9313 | N |  |
| 333 | *B. pauloensis* | Brasília | DF | 2012 | EFrançoso | LGEA | EF333 | frozen | -47.9804 | -15.9313 | N |  |
| 334 | *B. pauloensis* | Brasília | DF | 2012 | EFrançoso | LGEA | EF334 | frozen | -47.9804 | -15.9313 | N |  |
| 335 | *B. pauloensis* | Brasília | DF | 2012 | EFrançoso | LGEA | EF335 | frozen | -47.9804 | -15.9313 | N |  |
| 336 | *B. pauloensis* | Brasília | DF | 2012 | EFrançoso | LGEA | EF336 | frozen | -47.9804 | -15.9313 | N |  |
| 337 | *B. pauloensis* | Brasília | DF | 2012 | EFrançoso | LGEA | EF337 | frozen | -47.9804 | -15.9313 | N |  |
| 338 | *B. pauloensis* | Brasília | DF | 2012 | EFrançoso | LGEA | EF338 | frozen | -47.9804 | -15.9313 | N |  |
| 339 | *B. Pauloensis* | Brasília | DF | 2012 | EFrançoso | LGEA | EF339 | frozen | -47.8717 | -15.7614 | N |  |
| 340 | *B. pauloensis* | Brasília | DF | 2012 | EFrançoso | LGEA | EF340 | frozen | -47.8717 | -15.7614 | N | yes |
| 341 | *B. pauloensis* | Brasília | DF | 2012 | EFrançoso | LGEA | EF341 | frozen | -47.8717 | -15.7614 | N |  |
| 342 | *B. pauloensis* | Brasília | DF | 2012 | EFrançoso | LGEA | EF342 | frozen | -47.8717 | -15.7614 | N |  |
| 343 | *B. pauloensis* | Brasília | DF | 2012 | EFrançoso | LGEA | EF343 | frozen | -47.8717 | -15.7614 | N |  |
| 344 | *B. pauloensis* | Brasília | DF | 2012 | EFrançoso | LGEA | EF344 | frozen | -47.8717 | -15.7614 | N |  |
| 345 | *B. pauloensis* | Brasília | DF | 2012 | EFrançoso | LGEA | EF345 | frozen | -47.8717 | -15.7614 | N |  |
| 347 | *B. pauloensis* | Brasília | DF | 2012 | EFrançoso | LGEA | EF347 | frozen | -47.8717 | -15.7614 | N |  |
| 349 | *B. pauloensis* | Brasília | DF | 2012 | EFrançoso | LGEA | EF349 | frozen | -47.8717 | -15.7614 | N |  |
| 350 | *B. pauloensis* | Brasília | DF | 2012 | EFrançoso | LGEA | EF350 | frozen | -47.8717 | -15.7614 | N | yes |
| 352 | *B. pauloensis* | Brasília | DF | 2012 | EFrançoso | LGEA | EF352 | frozen | -47.8717 | -15.7614 | N |  |
| 353 | *B. pauloensis* | Brasília | DF | 2012 | EFrançoso | LGEA | EF353 | frozen | -47.8717 | -15.7614 | N |  |
| USP 11 | *B. pauloensis* | São Paulo | SP | 2009 | EFrançoso | LGEA | EFUSP 11 | frozen | -46.7312 | -23.5658 | N |  |
| USP 12 | *B. pauloensis* | São Paulo | SP | 2009 | EFrançoso | LGEA | EFUSP 12 | frozen | -46.7312 | -23.5658 | N |  |
| USP 2 | *B. pauloensis* | São Paulo | SP | 2009 | EFrançoso | LGEA | EFUSP 2 | frozen | -46.7312 | -23.5658 | N |  |
| USP 5 | *B. pauloensis* | São Paulo | SP | 2010 | EFrançoso | LGEA | EFUSP 5 | frozen | -46.7312 | -23.5658 | N |  |
| USP25 | *B. pauloensis* | São Paulo | SP | 2011 | EFrançoso | LGEA | EFUSP25 | frozen | -46.7312 | -23.5658 | N | yes |
| USP27 | *B. pauloensis* | São Paulo | SP | 2011 | EFrançoso | LGEA | EFUSP27 | frozen | -46.7312 | -23.5658 | N |  |
| USP28 | *B. pauloensis* | São Paulo | SP | 2011 | EFrançoso | LGEA | EFUSP28 | frozen | -46.7312 | -23.5658 | N |  |
| USP29 | *B. pauloensis* | São Paulo | SP | 2011 | EFrançoso | LGEA | EFUSP29 | frozen | -46.7312 | -23.5658 | N |  |
| USP34 | *B. pauloensis* | São Paulo | SP | 2011 | EFrançoso | LGEA | EFUSP34 | frozen | -46.7312 | -23.5658 | N |  |
| USP40 | *B. pauloensis* | São Paulo | SP | 2012 | WAugusto | LGEA | EFUSP40 | frozen | -46.7312 | -23.5658 | N |  |
| 14 | *B. pauloensis* | Apiaí | SP | 2009 | FOFrancisco | LGEA | FOF209 | frozen | -48.8432 | -24.5138 | S | yes |
| 17 | *B. pauloensis* | Apiaí | SP | 2009 | FOFrancisco | LGEA | FOF186 | frozen | -48.8432 | -24.5138 | S | yes |
| 43 | *B. pauloensis* | Prudentópolis | PR | 2010 | FOFrancisco | LGEA | EF43 | frozen | -50.9689 | -25.2155 | S | yes |
| 44 | *B. pauloensis* | Gonçalves | MG | 2007 | FASilveira | UFMG | 13453-39784 | pinned | -45.8544 | -22.6589 | S | yes |
| 49 | *B. pauloensis* | Painel | SC | 2007 | ASalino | UFMG | 12304-36244 | pinned | -50.1033 | -27.9250 | S | yes |
| 169 | *B. pauloensis* | Itaóca | SP | 2000 | FOFrancisco | LGEA | FOF169 | frozen | -47.1648 | -23.3555 | S | yes |
| 175 | *B. pauloensis* | Foz do Iguaçu | PR | 2011 | FOFrancisco | LGEA | EF175 | frozen | -53.4773 | -24.4495 | S | yes |
| 176 | *B. pauloensis* | Caçador | SC | 2011 | FOFrancisco | LGEA | EF176 | frozen | -50.9931 | -25.2123 | S | yes |
| 219 | *B. pauloensis* | Londrina | PR | 2005 |  | LGEA | EF219 | frozen | -51.1737 | -23.2961 | S | yes |
| 221 | *B. pauloensis* | Londrina | PR | 2011 |  | LGEA | EF221 | frozen | -51.1737 | -23.2961 | S | yes |
| 225 | *B. pauloensis* | Iporanga | SP | 2011 | FOFrancisco | LGEA | FOF370 | frozen | -47.3210 | -23.4472 | S |  |
| 229 | *B. pauloensis* | Iguape | SP | 2010 | FOFrancisco | LGEA | FOF804 | frozen | -46.4999 | -23.3611 | S | yes |
| 234 | *B. pauloensis* | Ilha comprida | SP | 2011 | FOFrancisco | LGEA | FOF831 | frozen | -46.4955 | -23.2790 | S | yes |
| 239 | *B. pauloensis* | Ilha comprida | SP | 2011 | FOFrancisco | LGEA | FOF839 | frozen | -46.4820 | -23.2850 | S |  |
| 240 | *B. pauloensis* | Ilha comprida | SP | 2011 | FOFrancisco | LGEA | FOF841 | frozen | -46.4820 | -23.2850 | S | yes |
| 241 | *B. pauloensis* | Ilha comprida | SP | 2011 | FOFrancisco | LGEA | FOF842 | frozen | -46.4820 | -23.2850 | S |  |
| 242 | *B. pauloensis* | Ilha comprida | SP | 2011 | FOFrancisco | LGEA | FOF844 | frozen | -46.4820 | -23.2850 | S |  |
| 244 | *B. pauloensis* | Iporanga | SP | 2011 | FOFrancisco | LGEA | FOF849 | frozen | -47.4158 | -23.4557 | S |  |
| 245 | *B. pauloensis* | Itaóca | SP | 2011 | FOFrancisco | LGEA | FOF850 | frozen | -47.1648 | -23.3555 | S | yes |
| 247 | *B. pauloensis* | Guaratuba | PR | 2011 | FOFrancisco | LGEA | FOF854 | frozen | -47.4030 | -24.0419 | S |  |
| 249 | *B. pauloensis* | Foz do Iguaçu | PR | 2011 | FOFrancisco | LGEA | FOF1419 | frozen | -53.4126 | -24.5278 | S | yes |
| 269 | *B. pauloensis* | Itapoá | SC | 2011 | FOFrancisco | LGEA | FOF1875 | frozen | -48.6171 | -26.1163 | S |  |
| 361 | *B. pauloensis* | Botuverá | SC | 2012 | EFrançoso | LGEA | EF361 | frozen | -49.1783 | -27.1969 | S | yes |
| 363 | *B. pauloensis* | Presidente Nereu | SC | 2013 | EFrançoso | LGEA | EF363 | frozen | -49.3296 | -27.2596 | S |  |
| 364 | *B. pauloensis* | Presidente Nereu | SC | 2013 | EFrançoso | LGEA | EF364 | frozen | -49.3296 | -27.2596 | S | yes |
| 365 | *B. pauloensis* | Presidente Nereu | SC | 2013 | EFrançoso | LGEA | EF365 | frozen | -49.3296 | -27.2596 | S |  |
| 366 | *B. pauloensis* | Presidente Nereu | SC | 2013 | EFrançoso | LGEA | EF366 | frozen | -49.3296 | -27.2596 | S |  |
| 367 | *B. pauloensis* | Presidente Nereu | SC | 2013 | EFrançoso | LGEA | EF367 | frozen | -49.3296 | -27.2596 | S |  |
| 368 | *B. pauloensis* | Presidente Nereu | SC | 2013 | EFrançoso | LGEA | EF368 | frozen | -49.3296 | -27.2596 | S |  |
| 370 | *B. pauloensis* | Lages | SC | 2013 | EFrançoso | LGEA | EF370 | frozen | -50.1917 | -27.4806 | S |  |
| 371 | *B. pauloensis* | Lages | SC | 2013 | EFrançoso | LGEA | EF371 | frozen | -50.1917 | -27.4806 | S |  |
| 372 | *B. pauloensis* | Lages | SC | 2013 | EFrançoso | LGEA | EF372 | frozen | -50.1917 | -27.4806 | S |  |
| 374 | *B. pauloensis* | Lages | SC | 2013 | EFrançoso | LGEA | EF374 | frozen | -50.1917 | -27.4806 | S |  |
| 375 | *B. pauloensis* | Lages | SC | 2013 | EFrançoso | LGEA | EF375 | frozen | -50.1917 | -27.4806 | S |  |
| 376 | *B. pauloensis* | Lages | SC | 2013 | EFrançoso | LGEA | EF376 | frozen | -50.1917 | -27.4806 | S | yes |
| 377 | *B. pauloensis* | Lages | SC | 2013 | EFrançoso | LGEA | EF377 | frozen | -50.1917 | -27.4806 | S |  |
| 378 | *B. pauloensis* | Lages | SC | 2013 | EFrançoso | LGEA | EF378 | frozen | -50.1917 | -27.4806 | S |  |
| 379 | *B. pauloensis* | Lages | SC | 2013 | EFrançoso | LGEA | EF379 | frozen | -50.1917 | -27.4806 | S |  |
| 380 | *B. pauloensis* | Lages | SC | 2013 | EFrançoso | LGEA | EF380 | frozen | -50.1958 | -27.4933 | S |  |
| 381 | *B. pauloensis* | Lages | SC | 2013 | EFrançoso | LGEA | EF381 | frozen | -50.1958 | -27.4933 | S |  |
| 382 | *B. pauloensis* | Lages | SC | 2013 | EFrançoso | LGEA | EF382 | frozen | -50.1958 | -27.4933 | S |  |
| 383 | *B. pauloensis* | Lages | SC | 2013 | EFrançoso | LGEA | EF383 | frozen | -50.1958 | -27.4933 | S |  |
| 384 | *B. pauloensis* | Lages | SC | 2013 | EFrançoso | LGEA | EF384 | frozen | -50.1958 | -27.4933 | S |  |
| 385 | *B. pauloensis* | Otacílio Costa | PR | 2013 | EFrançoso | LGEA | EF385 | frozen | -50.2003 | -27.4979 | S |  |
| 386 | *B. pauloensis* | Otacílio Costa | PR | 2013 | EFrançoso | LGEA | EF386 | frozen | -50.2003 | -27.4979 | S |  |
| 387 | *B. pauloensis* | Vacarias | RS | 2013 | EFrançoso | LGEA | EF387 | frozen | -50.5539 | -28.2995 | S | yes |
| 388 | *B. pauloensis* | Vacarias | RS | 2013 | EFrançoso | LGEA | EF388 | frozen | -50.5539 | -28.2995 | S |  |
| 389 | *B. pauloensis* | São Joaquim | SC | 2013 | EFrançoso | LGEA | EF389 | frozen | -50.5570 | -28.2984 | S | yes |
| 390 | *B. pauloensis* | Lages | SC | 2013 | EFrançoso | LGEA | EF390 | frozen | -50.5570 | -28.2984 | S | yes |
| 392 | *B. pauloensis* | Vacarias | RS | 2013 | EFrançoso | LGEA | EF392 | frozen | -51.0737 | -28.5439 | S |  |
| 394 | *B. pauloensis* | Caxias do Sul | RS | 2013 | EFrançoso | LGEA | EF394 | frozen | -51.2167 | -29.1238 | S | yes |
| 395 | *B. pauloensis* | Farroupilha | RS | 2013 | EFrançoso | LGEA | EF395 | frozen | -51.2168 | -29.1241 | S | yes |
| 396 | *B. pauloensis* | Farroupilha | RS | 2013 | EFrançoso | LGEA | EF396 | frozen | -51.2168 | -29.1241 | S |  |
| 397 | *B. pauloensis* | Farroupilha | RS | 2013 | EFrançoso | LGEA | EF397 | frozen | -51.2168 | -29.1241 | S |  |
| 398 | *B. pauloensis* | Farroupilha | RS | 2013 | EFrançoso | LGEA | EF398 | frozen | -51.2168 | -29.1241 | S |  |
| 399 | *B. pauloensis* | Farroupilha | RS | 2013 | EFrançoso | LGEA | EF399 | frozen | -51.2168 | -29.1241 | S |  |
| 400 | *B. pauloensis* | Farroupilha | RS | 2013 | EFrançoso | LGEA | EF400 | frozen | -51.2168 | -29.1241 | S |  |
| 401 | *B. pauloensis* | Farroupilha | RS | 2013 | EFrançoso | LGEA | EF401 | frozen | -51.2168 | -29.1241 | S |  |
| 402 | *B. pauloensis* | Farroupilha | RS | 2013 | EFrançoso | LGEA | EF402 | frozen | -51.2179 | -29.1344 | S |  |
| 403 | *B. pauloensis* | Caxias do Sul | RS | 2013 | EFrançoso | LGEA | EF403 | frozen | -51.2178 | -29.1344 | S |  |
| 404 | *B. pauloensis* | Caxias do Sul | RS | 2013 | EFrançoso | LGEA | EF404 | frozen | -51.2178 | -29.1344 | S | yes |
| 405 | *B. pauloensis* | Caxias do Sul | RS | 2013 | EFrançoso | LGEA | EF405 | frozen | -51.2178 | -29.1344 | S |  |
| 407 | *B. pauloensis* | Caxias do Sul | RS | 2013 | EFrançoso | LGEA | EF407 | frozen | -51.2178 | -29.1344 | S |  |
| 408 | *B. pauloensis* | Caxias do Sul | RS | 2013 | EFrançoso | LGEA | EF408 | frozen | -51.2178 | -29.1344 | S |  |
| 409 | *B. pauloensis* | Caxias do Sul | RS | 2013 | EFrançoso | LGEA | EF409 | frozen | -51.2178 | -29.1344 | S |  |
| 430 | *B. pauloensis* | Lajeado | RS | 2013 | EFrançoso | LGEA | EF430 | frozen | -52.0318 | -29.2312 | S | yes |
| 431 | *B. pauloensis* | Lajeado | RS | 2013 | EFrançoso | LGEA | EF431 | frozen | -52.0318 | -29.2312 | S |  |
| 446 | *B. pauloensis* | Santo Antônio do Planalto | RS | 2013 | EFrançoso | LGEA | EF446 | frozen | -52.4148 | -28.2399 | S | yes |
| 448 | *B. pauloensis* | Chapecó | SC | 2013 | EFrançoso | LGEA | EF448 | frozen | -52.3685 | -27.0744 | S | yes |
| 449 | *B. pauloensis* | Chapecó | SC | 2013 | EFrançoso | LGEA | EF449 | frozen | -52.3685 | -27.0744 | S | yes |
| 450 | *B. pauloensis* | Chapecó | SC | 2013 | EFrançoso | LGEA | EF450 | frozen | -52.3685 | -27.0744 | S | yes |
| 451 | *B. pauloensis* | Chapecó | SC | 2013 | EFrançoso | LGEA | EF451 | frozen | -52.3685 | -27.0744 | S | yes |
| 452 | *B. pauloensis* | Chapecó | SC | 2013 | EFrançoso | LGEA | EF452 | frozen | -52.3685 | -27.0744 | S | yes |
| 453 | *B. pauloensis* | Chapecó | SC | 2013 | EFrançoso | LGEA | EF453 | frozen | -52.3685 | -27.0744 | S |  |
| 454 | *B. pauloensis* | Chapecó | SC | 2013 | EFrançoso | LGEA | EF454 | frozen | -52.3685 | -27.0744 | S |  |
| 455 | *B. pauloensis* | Chapecó | SC | 2013 | EFrançoso | LGEA | EF455 | frozen | -52.3685 | -27.0744 | S |  |
| 456 | *B. pauloensis* | Chapecó | SC | 2013 | EFrançoso | LGEA | EF456 | frozen | -52.3685 | -27.0744 | S |  |
| 471 | *B. pauloensis* | Chapecó | SC | 2013 | EFrançoso | LGEA | EF471 | frozen | -52.3685 | -27.0744 | S |  |
| 474 | *B. pauloensis* | Chapecó | SC | 2013 | EFrançoso | LGEA | EF474 | frozen | -52.3674 | -27.0723 | S |  |
| 475 | *B. pauloensis* | Chapecó | SC | 2013 | EFrançoso | LGEA | EF475 | frozen | -52.3674 | -27.0723 | S |  |
| 476 | *B. pauloensis* | Chapecó | SC | 2013 | EFrançoso | LGEA | EF476 | frozen | -52.3674 | -27.0723 | S |  |
| 485 | *B. pauloensis* | São Mateus do Sul | PR | 2013 | EFrançoso | LGEA | EF485 | frozen | -50.4223 | -26.0252 | S |  |
| 487 | *B. pauloensis* | São Mateus do Sul | PR | 2013 | EFrançoso | LGEA | EF487 | frozen | -50.4223 | -26.0252 | S |  |
| 488 | *B. pauloensis* | São Mateus do Sul | PR | 2013 | EFrançoso | LGEA | EF488 | frozen | -50.4223 | -26.0252 | S | yes |
| 489 | *B. pauloensis* | São Mateus do Sul | PR | 2013 | EFrançoso | LGEA | EF489 | frozen | -50.4223 | -26.0252 | S |  |
| 490 | *B. pauloensis* | São Mateus do Sul | PR | 2013 | EFrançoso | LGEA | EF490 | frozen | -50.2390 | -25.5214 | S | yes |
| 491 | *B. pauloensis* | São Mateus do Sul | PR | 2013 | EFrançoso | LGEA | EF491 | frozen | -50.2319 | -25.2555 | S |  |
| 492 | *B. pauloensis* | São Mateus do Sul | PR | 2013 | EFrançoso | LGEA | EF492 | frozen | -50.2319 | -25.2555 | S |  |
| USP 10 | *B. pauloensis* | São Paulo | SP | 2009 | EFrançoso | LGEA | EFUSP 10 | frozen | -46.7312 | -23.5658 | S | yes |
| USP 3 | *B. pauloensis* | São Paulo | SP | 2009 | EFrançoso | LGEA | EFUSP 3 | frozen | -46.7312 | -23.5658 | S |  |
