# Supplementary material for: Comparative phylogeography in the Atlantic forest and Brazilian savannas: pleistocene fluctuations and dispersal shape spatial patterns in two bumblebees
Source: BMC Evol Biol. 2016 Dec 7;16:267. doi: 10.1186/s12862-016-0803-0 (PMC5142330; doi:10.1186/s12862-016-0803-0)
Supplement: Additional file 2: — Molecular clock calibration phylogeny from sequences and calibration points (arrows) used by Hines [39]. Through this phylogeny, the dating for the clade Thoracobombus (highlighted), the common ancestral node that includes Bombus morio and B. pauloensis, was estimated at 13,5962 Ma. This dating was used to calibrate the phylogeny for each one of these species, once B. pauloensis was used as the outgroup of B. morio and vice versa. (DOCX 80 kb) [file 12862_2016_803_MOESM2_ESM.docx]

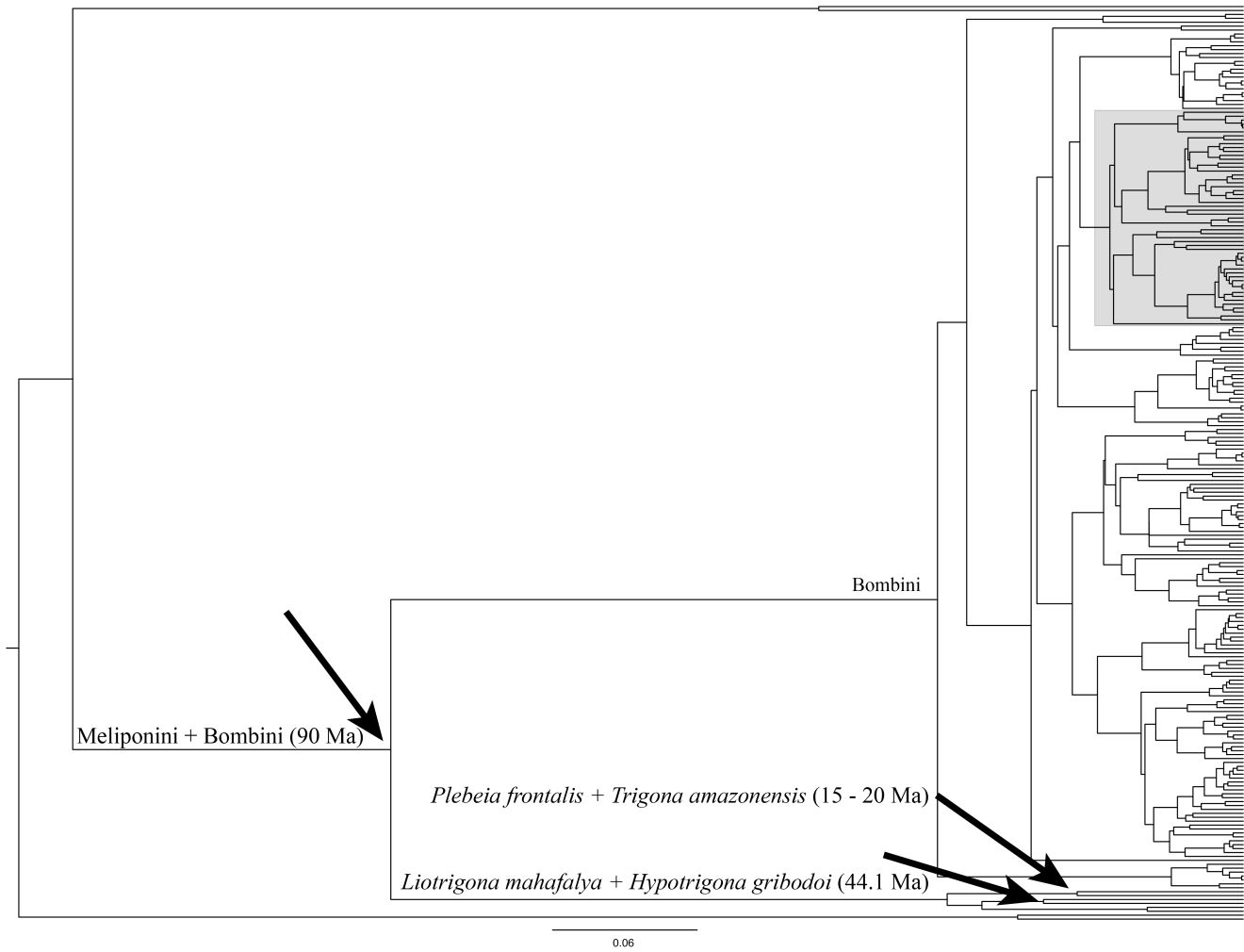


**Additional file 2** - Molecular clock calibration phylogeny from sequences and calibration points (arrows) used by Hines (2008). Through this phylogeny, the dating for the clade *Thoracobombus* (highlighted), the common ancestral node that includes *B. morio* and *B. pauloensis*, was estimated in 13.5962 Ma. This dating was used to calibrate the phylogeny for each one of these species, once *B. pauloensis* was used as outgroup of *B. morio* and vice versa.
