# Supplementary material for: Comparative phylogeography in the Atlantic forest and Brazilian savannas: pleistocene fluctuations and dispersal shape spatial patterns in two bumblebees
Source: BMC Evol Biol. 2016 Dec 7;16:267. doi: 10.1186/s12862-016-0803-0 (PMC5142330; doi:10.1186/s12862-016-0803-0)
Supplement: Additional file 3: — Genotypes results for Bombus morio. (DOCX 29 kb) [file 12862_2016_803_MOESM3_ESM.docx]

**Additional file 3** – Genotypes results for *B. morio.* The allele size (bp) per locus is presented

|  |  | **Loci** | | | | | | | | | | | | | | | |
| --- | --- | --- | --- | --- | --- | --- | --- | --- | --- | --- | --- | --- | --- | --- | --- | --- | --- |
| **Sample** | **Clade** | **BM1** | | **BM3** | | **BM4** | | **BM11** | | **BM13** | | **BM17** | | **BM18** | | **BM20** | |
| **15** | TS | 199 | 199 | 238 | 240 | 264 | 266 | 178 | 190 | 234 | 236 | 210 | 210 | 166 | 172 | 213 | 225 |
| **2** | Main | 197 | 199 | 240 | 242 | 264 | 266 | 168 | 180 | 228 | 230 | 212 | 212 | 170 | 182 | 189 | 207 |
| **10** | Main | 187 | 201 | 238 | 240 | 260 | 266 | 172 | 176 | 232 | 234 | 212 | 212 | 166 | 172 | 173 | 195 |
| **11** | Main | 199 | 201 | 220 | 238 | 262 | 266 | 170 | 180 | 230 | 234 | 212 | 228 | 170 | 172 | 185 | 201 |
| **13** | Main | 199 | 201 | 236 | 238 | 260 | 264 | 170 | 178 | 234 | 238 | 212 | 212 | 166 | 166 | 193 | 195 |
| **16** | Main | 203 | 203 | 218 | 238 | 252 | 260 | 154 | 158 | 230 | 234 | 214 | 218 | 180 | 182 | 201 | 215 |
| **22** | Main | 205 | 209 | 238 | 242 | 266 | 266 | 160 | 188 | 236 | 236 | 212 | 222 | 178 | 196 | 203 | 203 |
| **23** | Main | 197 | 207 | 236 | 240 | 262 | 264 | 152 | 154 | 228 | 228 | 228 | 232 | 174 | 174 | 199 | 205 |
| **24** | Main | 197 | 209 | 218 | 218 | 262 | 274 | 160 | 162 | 230 | 234 | 212 | 226 | 172 | 172 | 193 | 195 |
| **25** | Main | 211 | 211 | 238 | 238 | 260 | 266 | 190 | 196 | 232 | 236 | 212 | 220 | 186 | 190 | 193 | 217 |
| **28** | Main | 199 | 209 | 216 | 218 | 266 | 266 | 170 | 170 | 230 | 232 | 212 | 224 | 172 | 180 | 189 | 191 |
| **29** | Main | 199 | 207 | 260 | 264 | 262 | 266 | 188 | 188 | 232 | 232 | 212 | 222 | 168 | 174 | 189 | 189 |
| **31** | Main | 209 | 209 | 240 | 240 | 264 | 264 | 0 | 0 | 234 | 234 | 212 | 212 | 182 | 182 | 227 | 229 |
| **32** | Main | 203 | 203 | 236 | 236 | 262 | 270 | 0 | 0 | 230 | 234 | 212 | 212 | 170 | 172 | 193 | 201 |
| **33** | Main | 201 | 211 | 236 | 240 | 262 | 264 | 166 | 172 | 234 | 238 | 212 | 228 | 170 | 182 | 189 | 203 |
| **36** | Main | 195 | 197 | 218 | 228 | 262 | 266 | 166 | 170 | 234 | 238 | 212 | 236 | 184 | 186 | 191 | 209 |
| **37** | Main | 203 | 211 | 218 | 246 | 262 | 264 | 176 | 184 | 230 | 234 | 212 | 232 | 166 | 166 | 223 | 223 |
| **40** | Main | 209 | 209 | 238 | 238 | 264 | 264 | 166 | 166 | 230 | 230 | 230 | 230 | 178 | 178 | 215 | 217 |
| **42** | Main | 197 | 211 | 236 | 238 | 262 | 262 | 154 | 180 | 230 | 230 | 232 | 236 | 172 | 178 | 197 | 221 |
| **72** | Main | 205 | 207 | 240 | 242 | 262 | 268 | 176 | 180 | 230 | 232 | 212 | 220 | 180 | 192 | 193 | 205 |
| **84** | Main | 197 | 197 | 236 | 236 | 264 | 266 | 158 | 160 | 234 | 238 | 212 | 220 | 180 | 190 | 189 | 195 |
| **85** | Main | 199 | 207 | 218 | 240 | 262 | 266 | 170 | 182 | 234 | 234 | 212 | 212 | 160 | 174 | 195 | 203 |
| **86** | Main | 201 | 203 | 218 | 238 | 264 | 266 | 174 | 176 | 232 | 238 | 212 | 220 | 174 | 180 | 181 | 193 |
| **87** | Main | 199 | 199 | 218 | 240 | 262 | 266 | 176 | 176 | 234 | 238 | 212 | 212 | 172 | 190 | 189 | 195 |
| **88** | Main | 197 | 199 | 238 | 246 | 264 | 268 | 172 | 184 | 232 | 234 | 212 | 212 | 170 | 172 | 185 | 195 |
| **91** | Main | 201 | 201 | 220 | 236 | 262 | 268 | 176 | 178 | 230 | 236 | 212 | 212 | 166 | 178 | 195 | 199 |
| **95** | Main | 199 | 201 | 240 | 242 | 260 | 260 | 178 | 188 | 234 | 234 | 212 | 212 | 176 | 176 | 193 | 207 |
| **172** | Main | 197 | 205 | 218 | 220 | 260 | 260 | 172 | 182 | 234 | 234 | 212 | 212 | 172 | 178 | 189 | 195 |
| **174** | Main | 199 | 205 | 262 | 264 | 258 | 262 | 174 | 182 | 234 | 238 | 210 | 212 | 178 | 178 | 203 | 203 |
| **177** | Main | 199 | 199 | 264 | 264 | 232 | 260 | 174 | 182 | 232 | 236 | 212 | 212 | 172 | 174 | 205 | 205 |
| **180** | Main | 201 | 201 | 234 | 236 | 264 | 266 | 174 | 178 | 232 | 234 | 212 | 218 | 168 | 168 | 195 | 215 |
| **182** | Main | 199 | 201 | 216 | 236 | 264 | 272 | 180 | 184 | 234 | 250 | 212 | 218 | 166 | 174 | 191 | 195 |
| **184** | Main | 197 | 199 | 240 | 244 | 260 | 270 | 176 | 178 | 230 | 232 | 212 | 212 | 170 | 174 | 201 | 201 |
| **189** | Main | 201 | 201 | 218 | 242 | 266 | 272 | 160 | 168 | 234 | 234 | 212 | 212 | 170 | 172 | 195 | 195 |
| **190** | Main | 201 | 207 | 236 | 244 | 264 | 264 | 180 | 182 | 230 | 230 | 212 | 212 | 166 | 190 | 193 | 193 |
| **200** | Main | 199 | 199 | 238 | 240 | 262 | 270 | 178 | 182 | 238 | 238 | 212 | 226 | 172 | 176 | 193 | 203 |
| **201** | Main | 199 | 209 | 236 | 240 | 260 | 262 | 160 | 180 | 232 | 232 | 220 | 228 | 166 | 174 | 187 | 195 |
| **208** | Main | 197 | 203 | 234 | 236 | 262 | 266 | 170 | 172 | 230 | 238 | 212 | 218 | 170 | 174 | 207 | 207 |
| **217** | Main | 197 | 207 | 246 | 246 | 262 | 266 | 172 | 196 | 236 | 238 | 212 | 228 | 166 | 172 | 197 | 197 |
| **220** | Main | 197 | 199 | 236 | 244 | 262 | 266 | 166 | 174 | 234 | 238 | 212 | 238 | 172 | 172 | 199 | 199 |
| **222** | Main | 197 | 217 | 236 | 246 | 264 | 266 | 166 | 178 | 230 | 234 | 212 | 232 | 170 | 176 | 197 | 203 |
| **224** | Main | 197 | 199 | 236 | 240 | 262 | 264 | 158 | 180 | 230 | 230 | 212 | 220 | 172 | 176 | 193 | 199 |
| **321** | Main | 203 | 205 | 218 | 238 | 228 | 262 | 176 | 192 | 230 | 234 | 212 | 220 | 166 | 176 | 185 | 187 |
| **354** | Main | 199 | 203 | 244 | 244 | 262 | 264 | 176 | 184 | 230 | 234 | 212 | 218 | 172 | 174 | 195 | 203 |
| **355** | Main | 197 | 205 | 238 | 238 | 266 | 276 | 176 | 180 | 234 | 234 | 212 | 212 | 172 | 172 | 187 | 197 |
| **358** | Main | 195 | 205 | 218 | 238 | 264 | 276 | 180 | 196 | 230 | 234 | 220 | 220 | 166 | 172 | 191 | 195 |
| **362** | Main | 203 | 211 | 238 | 238 | 264 | 268 | 152 | 154 | 234 | 234 | 212 | 232 | 166 | 170 | 183 | 185 |
| **369** | Main | 201 | 201 | 238 | 244 | 264 | 264 | 154 | 178 | 234 | 234 | 212 | 234 | 196 | 196 | 193 | 211 |
| **391** | Main | 209 | 213 | 238 | 238 | 262 | 262 | 154 | 182 | 236 | 236 | 212 | 238 | 172 | 174 | 187 | 195 |
| **412** | Main | 197 | 203 | 236 | 240 | 258 | 264 | 166 | 172 | 234 | 238 | 212 | 212 | 172 | 184 | 185 | 197 |
| **421** | Main | 203 | 209 | 236 | 238 | 260 | 266 | 182 | 182 | 230 | 234 | 212 | 212 | 166 | 168 | 191 | 205 |
| **423** | Main | 203 | 203 | 236 | 244 | 264 | 266 | 170 | 178 | 234 | 234 | 210 | 212 | 168 | 174 | 183 | 189 |
| **435** | Main | 209 | 213 | 218 | 236 | 266 | 268 | 182 | 190 | 236 | 238 | 212 | 222 | 184 | 186 | 207 | 209 |
| **440** | Main | 205 | 207 | 238 | 242 | 262 | 264 | 158 | 170 | 236 | 236 | 210 | 212 | 166 | 168 | 207 | 209 |
| **442** | Main | 197 | 199 | 238 | 240 | 260 | 266 | 166 | 174 | 232 | 234 | 224 | 238 | 172 | 184 | 187 | 197 |
| **447** | Main | 201 | 203 | 236 | 238 | 264 | 264 | 176 | 184 | 234 | 234 | 210 | 212 | 170 | 176 | 191 | 199 |
| **459** | Main | 201 | 211 | 238 | 244 | 264 | 266 | 162 | 172 | 234 | 234 | 212 | 224 | 170 | 174 | 0 | 0 |
| **460** | Main | 199 | 209 | 218 | 244 | 234 | 266 | 170 | 170 | 234 | 238 | 212 | 212 | 170 | 172 | 189 | 205 |
| **461** | Main | 201 | 201 | 236 | 242 | 258 | 262 | 162 | 180 | 234 | 238 | 220 | 224 | 166 | 172 | 187 | 189 |
| **462** | Main | 209 | 211 | 220 | 238 | 262 | 266 | 158 | 172 | 232 | 234 | 212 | 234 | 176 | 186 | 189 | 197 |
| **463** | Main | 197 | 203 | 218 | 238 | 266 | 266 | 154 | 184 | 234 | 234 | 218 | 226 | 166 | 170 | 207 | 209 |
| **464** | Main | 199 | 201 | 218 | 242 | 266 | 268 | 154 | 178 | 230 | 232 | 210 | 226 | 172 | 186 | 197 | 197 |
| **486** | Main | 211 | 199 | 218 | 220 | 264 | 266 | 170 | 178 | 234 | 234 | 212 | 212 | 166 | 172 | 187 | 189 |
| **496** | Main | 209 | 209 | 240 | 240 | 268 | 268 | 166 | 188 | 232 | 236 | 212 | 232 | 170 | 180 | 191 | 221 |
| **498** | Main | 201 | 205 | 218 | 240 | 262 | 270 | 154 | 178 | 232 | 234 | 218 | 226 | 180 | 182 | 194 | 194 |
| **USP18** | Main | 197 | 209 | 242 | 242 | 260 | 266 | 164 | 178 | 230 | 234 | 210 | 210 | 166 | 170 | 179 | 207 |
| **USP22** | Main | 195 | 195 | 236 | 236 | 264 | 264 | 178 | 178 | 234 | 234 | 212 | 212 | 172 | 172 | 187 | 187 |
