# Supplementary material for: Comparative phylogeography in the Atlantic forest and Brazilian savannas: pleistocene fluctuations and dispersal shape spatial patterns in two bumblebees
Source: BMC Evol Biol. 2016 Dec 7;16:267. doi: 10.1186/s12862-016-0803-0 (PMC5142330; doi:10.1186/s12862-016-0803-0)
Supplement: Additional file 4: — Genotypes results for Bombus pauloensis. (DOCX 35 kb) [file 12862_2016_803_MOESM4_ESM.docx]

**Additional file 4** – Genotypes results for *B. pauloensis.* The allele size (bp) per locus is presented

|  |  | **Loci** | | | | | | | | | | | | | | | |
| --- | --- | --- | --- | --- | --- | --- | --- | --- | --- | --- | --- | --- | --- | --- | --- | --- | --- |
| **Sample** | **Clade** | **BA2** | | **BA9** | | **BA11** | | **BA15** | | **BA8** | | **BA4** | | **BA7** | | **BA17** | |
| **235** | C | 234 | 234 | 114 | 124 | 196 | 200 | 254 | 254 | 210 | 220 | 118 | 136 | 221 | 221 | 190 | 190 |
| **246** | C | 230 | 240 | 122 | 124 | 196 | 212 | 252 | 254 | 210 | 212 | 118 | 124 | 215 | 221 | 208 | 222 |
| **259** | C | 230 | 230 | 112 | 130 | 198 | 198 | 256 | 260 | 210 | 222 | 120 | 120 | 201 | 217 | 186 | 192 |
| **266** | C | 222 | 230 | 114 | 122 | 200 | 202 | 254 | 264 | 210 | 214 | 116 | 124 | 211 | 223 | 190 | 204 |
| **170** | C | 230 | 230 | 128 | 128 | 198 | 224 | 254 | 254 | 210 | 228 | 122 | 160 | 211 | 219 | 186 | 204 |
| **230** | C | 230 | 230 | 114 | 114 | 194 | 196 | 252 | 254 | 212 | 216 | 122 | 122 | 223 | 239 | 176 | 202 |
| **231** | C | 216 | 228 | 126 | 128 | 198 | 220 | 254 | 254 | 210 | 222 | 120 | 124 | 217 | 217 | 190 | 198 |
| **232** | C | 226 | 240 | 126 | 132 | 192 | 196 | 252 | 254 | 206 | 222 | 120 | 128 | 225 | 225 | 176 | 176 |
| **233** | C | 230 | 234 | 124 | 126 | 0 | 0 | 252 | 256 | 226 | 228 | 124 | 132 | 211 | 217 | 198 | 202 |
| **236** | C | 230 | 230 | 114 | 114 | 196 | 198 | 258 | 262 | 210 | 212 | 118 | 122 | 217 | 219 | 202 | 206 |
| **237** | C | 230 | 230 | 114 | 128 | 196 | 198 | 252 | 254 | 212 | 234 | 116 | 126 | 217 | 217 | 190 | 194 |
| **238** | C | 228 | 236 | 114 | 128 | 196 | 198 | 254 | 260 | 210 | 214 | 118 | 122 | 211 | 211 | 176 | 206 |
| **243** | C | 236 | 240 | 114 | 128 | 196 | 244 | 254 | 260 | 210 | 216 | 118 | 122 | 209 | 211 | 176 | 206 |
| **248** | C | 230 | 240 | 122 | 124 | 200 | 214 | 252 | 260 | 210 | 212 | 120 | 124 | 215 | 225 | 208 | 222 |
| **268** | C | 230 | 230 | 120 | 120 | 194 | 194 | 254 | 254 | 210 | 214 | 118 | 136 | 211 | 223 | 190 | 202 |
| **270** | C | 230 | 232 | 122 | 132 | 196 | 198 | 258 | 258 | 212 | 222 | 122 | 124 | 227 | 233 | 198 | 214 |
| **271** | C | 220 | 228 | 122 | 128 | 194 | 198 | 256 | 260 | 210 | 210 | 120 | 122 | 217 | 219 | 178 | 188 |
| **272** | C | 220 | 228 | 114 | 126 | 196 | 200 | 254 | 260 | 214 | 228 | 126 | 126 | 217 | 225 | 188 | 190 |
| **273** | C | 228 | 228 | 114 | 128 | 196 | 224 | 254 | 256 | 210 | 212 | 122 | 124 | 211 | 227 | 200 | 206 |
| **274** | C | 230 | 230 | 122 | 128 | 194 | 196 | 254 | 254 | 210 | 218 | 126 | 126 | 223 | 225 | 188 | 196 |
| **277** | C | 230 | 236 | 130 | 134 | 222 | 222 | 254 | 260 | 224 | 224 | 124 | 128 | 223 | 227 | 204 | 204 |
| **9** | N | 230 | 230 | 114 | 124 | 194 | 194 | 254 | 254 | 224 | 226 | 118 | 134 | 211 | 219 | 188 | 194 |
| **45** | N | 232 | 232 | 124 | 124 | 194 | 194 | 256 | 256 | 212 | 212 | 116 | 116 | 231 | 233 | 198 | 200 |
| **47** | N | 216 | 216 | 126 | 126 | 196 | 196 | 256 | 256 | 216 | 216 | 128 | 128 | 217 | 217 | 198 | 198 |
| **51** | N | 216 | 216 | 114 | 114 | 194 | 194 | 254 | 254 | 220 | 220 | 124 | 124 | 211 | 211 | 202 | 204 |
| **52** | N | 228 | 228 | 130 | 130 | 198 | 198 | 254 | 254 | 214 | 214 | 118 | 118 | 235 | 235 | 198 | 200 |
| **53** | N | 216 | 216 | 112 | 112 | 194 | 194 | 252 | 252 | 216 | 222 | 124 | 150 | 211 | 211 | 200 | 206 |
| **55** | N | 240 | 240 | 114 | 124 | 192 | 198 | 254 | 254 | 208 | 220 | 118 | 148 | 211 | 219 | 188 | 198 |
| **56** | N | 230 | 230 | 122 | 128 | 192 | 192 | 250 | 254 | 216 | 220 | 128 | 132 | 211 | 225 | 198 | 200 |
| **57** | N | 230 | 230 | 128 | 128 | 194 | 194 | 254 | 254 | 220 | 220 | 122 | 122 | 215 | 215 | 198 | 200 |
| **58** | N | 230 | 232 | 112 | 128 | 194 | 194 | 256 | 256 | 220 | 220 | 118 | 128 | 223 | 223 | 200 | 216 |
| **62** | N | 230 | 230 | 114 | 114 | 194 | 198 | 254 | 262 | 212 | 228 | 118 | 132 | 231 | 235 | 186 | 204 |
| **66** | N | 230 | 230 | 114 | 114 | 194 | 194 | 254 | 254 | 224 | 224 | 122 | 122 | 219 | 219 | 198 | 198 |
| **68** | N | 230 | 230 | 132 | 132 | 194 | 200 | 256 | 264 | 210 | 222 | 118 | 126 | 223 | 227 | 176 | 198 |
| **105** | N | 230 | 230 | 114 | 114 | 194 | 194 | 254 | 254 | 214 | 214 | 118 | 118 | 215 | 215 | 176 | 176 |
| **110** | N | 228 | 230 | 112 | 126 | 194 | 194 | 254 | 262 | 204 | 220 | 118 | 118 | 201 | 237 | 176 | 200 |
| **111** | N | 232 | 236 | 114 | 130 | 194 | 194 | 250 | 254 | 208 | 218 | 116 | 124 | 211 | 217 | 200 | 204 |
| **135** | N | 230 | 230 | 114 | 130 | 196 | 202 | 252 | 254 | 222 | 226 | 104 | 118 | 225 | 227 | 176 | 216 |
| **122** | N | 232 | 232 | 124 | 126 | 200 | 200 | 250 | 254 | 216 | 216 | 124 | 126 | 217 | 237 | 198 | 200 |
| **124** | N | 230 | 230 | 114 | 114 | 198 | 198 | 258 | 258 | 214 | 214 | 118 | 118 | 211 | 211 | 198 | 200 |
| **126** | N | 230 | 230 | 126 | 126 | 198 | 198 | 266 | 266 | 222 | 222 | 118 | 118 | 217 | 217 | 204 | 206 |
| **132** | N | 224 | 230 | 114 | 124 | 194 | 196 | 254 | 262 | 208 | 228 | 132 | 154 | 217 | 223 | 198 | 214 |
| **133** | N | 228 | 230 | 118 | 124 | 194 | 196 | 254 | 260 | 214 | 234 | 124 | 126 | 221 | 223 | 204 | 206 |
| **178** | N | 230 | 232 | 126 | 130 | 196 | 198 | 254 | 256 | 210 | 220 | 114 | 120 | 217 | 221 | 176 | 214 |
| **179** | N | 230 | 230 | 128 | 128 | 192 | 196 | 252 | 260 | 204 | 212 | 116 | 128 | 217 | 217 | 200 | 212 |
| **194** | N | 230 | 232 | 112 | 124 | 194 | 194 | 254 | 264 | 218 | 222 | 124 | 128 | 211 | 223 | 188 | 218 |
| **198** | N | 216 | 240 | 124 | 132 | 194 | 198 | 250 | 250 | 222 | 228 | 126 | 126 | 211 | 233 | 198 | 200 |
| **223** | N | 230 | 230 | 114 | 134 | 194 | 194 | 252 | 260 | 208 | 226 | 116 | 124 | 225 | 235 | 186 | 200 |
| **226** | N | 230 | 234 | 114 | 130 | 194 | 194 | 254 | 256 | 210 | 220 | 120 | 126 | 213 | 223 | 176 | 202 |
| **250** | N | 228 | 230 | 112 | 132 | 194 | 194 | 254 | 256 | 216 | 228 | 118 | 120 | 211 | 211 | 202 | 204 |
| **252** | N | 228 | 228 | 112 | 122 | 194 | 196 | 250 | 254 | 212 | 218 | 122 | 124 | 217 | 223 | 200 | 200 |
| **253** | N | 230 | 230 | 112 | 116 | 194 | 196 | 254 | 262 | 204 | 208 | 118 | 122 | 223 | 231 | 196 | 200 |
| **254** | N | 242 | 246 | 126 | 134 | 192 | 194 | 254 | 254 | 212 | 228 | 118 | 130 | 213 | 217 | 174 | 198 |
| **263** | N | 228 | 230 | 114 | 128 | 194 | 196 | 256 | 258 | 208 | 210 | 116 | 122 | 223 | 229 | 176 | 176 |
| **322** | N | 230 | 230 | 126 | 128 | 194 | 194 | 258 | 262 | 204 | 216 | 118 | 124 | 223 | 227 | 176 | 192 |
| **323** | N | 216 | 230 | 124 | 128 | 194 | 194 | 254 | 254 | 212 | 214 | 122 | 128 | 217 | 217 | 198 | 200 |
| **324** | N | 230 | 232 | 136 | 136 | 194 | 196 | 254 | 270 | 212 | 214 | 122 | 122 | 215 | 223 | 204 | 210 |
| **325** | N | 230 | 240 | 114 | 130 | 194 | 196 | 254 | 256 | 220 | 246 | 124 | 130 | 221 | 225 | 202 | 208 |
| **340** | N | 222 | 230 | 114 | 114 | 194 | 194 | 254 | 258 | 230 | 232 | 118 | 120 | 219 | 237 | 180 | 196 |
| **350** | N | 222 | 226 | 116 | 120 | 198 | 198 | 254 | 254 | 216 | 224 | 116 | 122 | 217 | 223 | 198 | 204 |
| **USP25** | N | 230 | 230 | 116 | 132 | 194 | 194 | 212 | 220 | 208 | 220 | 118 | 124 | 225 | 231 | 176 | 204 |
| **54** | N | 230 | 230 | 124 | 126 | 194 | 194 | 252 | 252 | 216 | 220 | 118 | 160 | 211 | 223 | 190 | 206 |
| **14** | S | 230 | 230 | 116 | 124 | 194 | 198 | 254 | 256 | 212 | 218 | 116 | 140 | 221 | 227 | 202 | 206 |
| **17** | S | 230 | 232 | 112 | 124 | 194 | 196 | 254 | 264 | 212 | 220 | 118 | 126 | 221 | 237 | 196 | 206 |
| **43** | S | 230 | 232 | 128 | 128 | 198 | 218 | 262 | 264 | 216 | 216 | 116 | 130 | 211 | 227 | 174 | 200 |
| **44** | S | 230 | 230 | 114 | 128 | 194 | 194 | 256 | 260 | 212 | 226 | 124 | 124 | 221 | 223 | 190 | 200 |
| **49** | S | 234 | 238 | 116 | 116 | 196 | 214 | 252 | 254 | 210 | 212 | 116 | 120 | 213 | 225 | 200 | 202 |
| **169** | S | 230 | 238 | 112 | 120 | 228 | 250 | 252 | 260 | 212 | 228 | 116 | 116 | 213 | 219 | 200 | 204 |
| **175** | S | 230 | 230 | 124 | 124 | 196 | 196 | 258 | 258 | 224 | 224 | 124 | 124 | 231 | 233 | 200 | 200 |
| **176** | S | 230 | 232 | 114 | 132 | 214 | 224 | 254 | 254 | 218 | 220 | 118 | 118 | 211 | 231 | 176 | 176 |
| **219** | S | 230 | 230 | 124 | 126 | 196 | 212 | 252 | 254 | 214 | 220 | 118 | 126 | 211 | 227 | 200 | 202 |
| **221** | S | 230 | 232 | 126 | 130 | 194 | 196 | 260 | 262 | 214 | 224 | 124 | 130 | 211 | 217 | 198 | 202 |
| **245** | S | 238 | 238 | 134 | 134 | 196 | 196 | 258 | 258 | 214 | 214 | 124 | 124 | 227 | 227 | 186 | 186 |
| **249** | S | 230 | 230 | 112 | 126 | 222 | 222 | 254 | 262 | 206 | 218 | 124 | 126 | 211 | 219 | 200 | 200 |
| **361** | S | 230 | 230 | 126 | 128 | 194 | 196 | 254 | 254 | 210 | 224 | 122 | 126 | 213 | 223 | 202 | 202 |
| **364** | S | 228 | 230 | 112 | 132 | 194 | 194 | 260 | 264 | 188 | 210 | 116 | 118 | 211 | 213 | 188 | 200 |
| **376** | S | 230 | 230 | 114 | 124 | 198 | 198 | 254 | 260 | 216 | 222 | 118 | 122 | 221 | 223 | 198 | 200 |
| **387** | S | 230 | 232 | 112 | 122 | 198 | 222 | 254 | 260 | 212 | 214 | 124 | 152 | 231 | 247 | 176 | 202 |
| **389** | S | 242 | 242 | 120 | 126 | 198 | 222 | 0 | 0 | 210 | 212 | 160 | 160 | 213 | 215 | 190 | 192 |
| **390** | S | 230 | 232 | 112 | 116 | 198 | 202 | 262 | 266 | 210 | 222 | 122 | 122 | 225 | 227 | 176 | 198 |
| **394** | S | 216 | 240 | 124 | 126 | 238 | 238 | 254 | 254 | 210 | 210 | 122 | 130 | 223 | 223 | 194 | 194 |
| **395** | S | 230 | 230 | 112 | 116 | 224 | 234 | 254 | 254 | 218 | 224 | 120 | 122 | 227 | 243 | 198 | 216 |
| **404** | S | 230 | 230 | 124 | 124 | 226 | 238 | 262 | 262 | 210 | 214 | 126 | 138 | 219 | 233 | 174 | 198 |
| **430** | S | 230 | 230 | 128 | 132 | 198 | 200 | 254 | 258 | 204 | 214 | 120 | 140 | 211 | 213 | 206 | 208 |
| **446** | S | 230 | 230 | 116 | 130 | 202 | 220 | 212 | 224 | 212 | 214 | 116 | 130 | 223 | 225 | 176 | 198 |
| **448** | S | 230 | 230 | 130 | 130 | 220 | 220 | 260 | 260 | 214 | 214 | 118 | 118 | 233 | 233 | 194 | 194 |
| **449** | S | 230 | 230 | 114 | 126 | 200 | 200 | 256 | 256 | 216 | 220 | 0 | 0 | 213 | 213 | 200 | 204 |
| **450** | S | 230 | 230 | 126 | 126 | 198 | 206 | 252 | 262 | 210 | 214 | 120 | 122 | 211 | 231 | 176 | 200 |
| **451** | S | 230 | 234 | 112 | 126 | 196 | 208 | 252 | 256 | 210 | 212 | 132 | 150 | 213 | 229 | 202 | 216 |
| **452** | S | 230 | 230 | 114 | 128 | 222 | 232 | 252 | 264 | 210 | 228 | 122 | 122 | 227 | 243 | 194 | 206 |
| **488** | S | 230 | 240 | 114 | 126 | 196 | 198 | 254 | 268 | 210 | 222 | 124 | 126 | 219 | 233 | 196 | 200 |
| **490** | S | 230 | 230 | 114 | 134 | 236 | 238 | 254 | 254 | 214 | 214 | 118 | 122 | 223 | 227 | 198 | 202 |
| **USP10** | S | 230 | 230 | 126 | 132 | 194 | 194 | 254 | 258 | 216 | 222 | 124 | 134 | 219 | 221 | 176 | 202 |
| **229** | S | 232 | 240 | 114 | 126 | 186 | 202 | 254 | 254 | 210 | 220 | 124 | 160 | 205 | 217 | 176 | 194 |
| **234** | S | 230 | 240 | 124 | 124 | 228 | 240 | 256 | 260 | 206 | 210 | 122 | 140 | 217 | 227 | 206 | 206 |
| **240** | S | 228 | 230 | 114 | 126 | 192 | 198 | 254 | 254 | 214 | 220 | 116 | 118 | 211 | 227 | 198 | 202 |
