# Supplementary material for: Comparative phylogeography in the Atlantic forest and Brazilian savannas: pleistocene fluctuations and dispersal shape spatial patterns in two bumblebees
Source: BMC Evol Biol. 2016 Dec 7;16:267. doi: 10.1186/s12862-016-0803-0 (PMC5142330; doi:10.1186/s12862-016-0803-0)
Supplement: Additional file 5: — Georeferenced occurrence records for Bombus morio and B. pauloensis used for geographic distribution modeling. (DOCX 35 kb) [file 12862_2016_803_MOESM5_ESM.docx]

**Additional file 5** - Georeferenced occurrence records for *B. morio* and *B. pauloensis* used for Geographic distribution modeling

| **Species** | **Collect City** | **State** | **Longitude** | **Latitude** |
| --- | --- | --- | --- | --- |
| *B. morio* | Conceição do Almeida | BA | -39.17000000 | -12.7794 |
| *B. morio* | Milagres | BA | -39.85890000 | -12.87 |
| *B. morio* | Lençóis | BA | -41.71666667 | -13.81666944 |
| *B. morio* | Igrapiúna | BA | -39.16478600 | -13.68183 |
| *B. morio* | Brasília | DF | -47.92913889 | -15.78016111 |
| *B. morio* | Dores do Rio Preto | ES | -41.84176111 | -20.69324444 |
| *B. morio* | Guarapari | ES | -40.50487300 | -20.652443 |
| *B. morio* | Alto Paraíso de Goiás | GO | -47.52151389 | -14.13355833 |
| *B. morio* | Goiás | GO | -50.14152778 | -15.93313889 |
| *B. morio* | Aiuruoca | MG | -44.60193611 | -21.92937778 |
| *B. morio* | Belo Horizonte | MG | -43.96573056 | -19.81573056 |
| *B. morio* | Bocaiúva | MG | -43.82121944 | -17.11515556 |
| *B. morio* | Brasilândia de Minas | MG | -46.01522500 | -17.01936667 |
| *B. morio* | Chapada Gaúcha | MG | -45.41825833 | -15.46842222 |
| *B. morio* | Curvelo | MG | -44.44675556 | -18.74916667 |
| *B. morio* | Divinópolis | MG | -44.88718611 | -20.13943611 |
| *B. morio* | Grão Mogol | MG | -42.89388611 | -16.55746667 |
| *B. morio* | Ibirité | MG | -44.05647222 | -20.01948333 |
| *B. morio* | Itacambira | MG | -43.31154444 | -17.06403611 |
| *B. morio* | itamarandiba | MG | -42.86096667 | -17.85665833 |
| *B. morio* | Itamonte | MG | -44.86801389 | -22.28900556 |
| *B. morio* | Joaquim Felício | MG | -44.14634722 | -17.77965833 |
| *B. morio* | Lavras | MG | -44.99977222 | -21.24574167 |
| *B. morio* | Lima Duarte | MG | -43.80753333 | -21.84834722 |
| *B. morio* | Serra do Cipó | MG | -43.65006500 | -19.000068 |
| *B. morio* | Ouro Preto | MG | -43.50366111 | -20.38553889 |
| *B. morio* | Passos | MG | -46.61007778 | -20.720375 |
| *B. morio* | Sabará | MG | -43.80482222 | -19.88918056 |
| *B. morio* | Salto da Divisa | MG | -39.94810278 | -16.01599167 |
| *B. morio* | Santana do Riacho | MG | -43.68025833 | -19.11704167 |
| *B. morio* | São Gonçalo do Rio Abaixo | MG | -43.38198333 | -19.82806944 |
| *B. morio* | São Gonçalo do Rio Preto | MG | -43.39526389 | -18.00662222 |
| *B. morio* | Taiobeiras | MG | -42.24164444 | -15.803625 |
| *B. morio* | Tiradentes | MG | -44.16850833 | -21.11130833 |
| *B. morio* | Três Marias | MG | -45.23262222 | -18.20503333 |
| *B. morio* | Uberlândia | MG | -48.26219167 | -18.91130556 |
| *B. morio* | Araguari | MG | -48.18287000 | -18.64745 |
| *B. morio* | Uberlândia | MG | -48.28550000 | -18.94397222 |
| *B. morio* | Céu Azul | PR | -53.84557778 | -25.14977778 |
| *B. morio* | Lindoeste | PR | -53.57609444 | -25.25915833 |
| *B. morio* | Morretes | PR | -48.83158889 | -25.47901389 |
| *B. morio* | Ponta Grossa | PR | -50.16321600 | -25.094644 |
| *B. morio* | Santa Lúcia | PR | -53.57058056 | -25.41101667 |
| *B. morio* | Santa Maria | PR | -52.91666667 | -26.33333333 |
| *B. morio* | Santa Tereza do Oeste | PR | -53.62758056 | -25.05270278 |
| *B. morio* | Santa Terezinha de Itaipu | PR | -54.40709722 | -25.44887222 |
| *B. morio* | Santa Lúcia | PR | -53.57058056 | -25.41101667 |
| *B. morio* | Céu Azul | PR | -53.84557778 | -25.14977778 |
| *B. morio* | Santa Maria | PR | -52.91666667 | -26.33333333 |
| *B. morio* | Céu Azul | PR | -53.84557778 | -25.14977778 |
| *B. morio* | Lindoeste | PR | -53.57609444 | -25.25915833 |
| *B. morio* | Santa Terezinha do Itaipu | PR | -54.40709722 | -25.44887222 |
| *B. morio* | Londrina | PR | -51.17366500 | -23.296075 |
| *B. morio* | São Mateus do Sul | PR | -50.42231000 | -26.02515 |
| *B. morio* | Angra dos Reis | RJ | -44.31838333 | -23.01005 |
| *B. morio* | Petrópolis | RJ | -43.18263611 | -22.504775 |
| *B. morio* | Serra dos Órgãos | RJ | -42.64609800 | -22.363013 |
| *B. morio* | Teresópolis | RJ | -42.96643333 | -22.412325 |
| *B. morio* | Angra dos Reis | RJ | -44.31838333 | -23.01005 |
| *B. morio* | Petrópolis | RJ | -43.18263611 | -22.504775 |
| *B. morio* | Nova Petrópolis | RS | -51.11847000 | -29.383224 |
| *B. morio* | Viamão | RS | -51.02378333 | -30.08861111 |
| *B. morio* | Vacarias | RS | -51.07365000 | 28.54387 |
| *B. morio* | Caxias do Sul | RS | -51.21782000 | -29.13437 |
| *B. morio* | Lajeado | RS | -52.03175000 | -29.23119 |
| *B. morio* | Santo Antônio do Planalto | RS | -52.41477000 | -28.23991 |
| *B. morio* | Presidente Nereu | SC | -49.32964000 | -27.25963 |
| *B. morio* | Chapecó | SC | -52.36847000 | -27.07444 |
| *B. morio* | Agudos | SP | -48.98941944 | -22.46914167 |
| *B. morio* | Apiaí | SP | -48.84320556 | -24.51376667 |
| *B. morio* | Atibaia | SP | -46.55030000 | -23.1169 |
| *B. morio* | Cajuru | SP | -47.30426667 | -21.27576944 |
| *B. morio* | Campinas | SP | -47.06080000 | -22.9056 |
| *B. morio* | Cotia | SP | -46.91920000 | -23.6039 |
| *B. morio* | Guaratuba | SP | -46.12852222 | -23.86073333 |
| *B. morio* | Ibiúna | SP | -47.22573333 | -23.65686111 |
| *B. morio* | Iguape | SP | -47.55530000 | -24.7081 |
| *B. morio* | Ilha Bela | SP | -45.32388611 | -23.917075 |
| *B. morio* | Luís Antônio | SP | -47.70440000 | -21.555 |
| *B. morio* | Piracicaba | SP | -47.65121389 | -22.72563889 |
| *B. morio* | Pirassununga | SP | -47.42691667 | -21.99611667 |
| *B. morio* | Ribeirão Preto | SP | -47.82111111 | -21.17659167 |
| *B. morio* | Santa Rita do Passa Quatro | SP | -47.47810000 | -21.7103 |
| *B. morio* | Santa Rosa do Viterbo | SP | -47.36220000 | -21.47063333 |
| *B. morio* | Santo josé do Barreiro | SP | -44.57529444 | -22.64537778 |
| *B. morio* | São Paulo | SP | -46.60456667 | -23.60522222 |
| *B. morio* | São Sebastião | SP | -45.41052778 | -23.758975 |
| *B. morio* | Teodoro Sampaio | SP | -52.18535833 | -22.53196667 |
| *B. morio* | Ubatuba | SP | -45.07110000 | -23.4339 |
| *B. morio* | Teodoro Sampaio | SP | -52.18535833 | -22.53196667 |
| *B. morio* | Apiaí | SP | -48.84320556 | -24.51376667 |
| *B. morio* | Ilha Bela | SP | -45.32388611 | -23.917075 |
| *B. morio* | Guaratuba | SP | -46.12852222 | -23.86073333 |
| *B. morio* | Ibiúna | SP | -47.22573333 | -23.65686111 |
| *B. morio* | Santo Antonio do Barreiro | SP | -44.57529444 | -22.64537778 |
| *B. morio* | Cajuru | SP | -47.30426667 | -21.27576944 |
| *B. morio* | Ribeirão Preto | SP | -47.82111111 | -21.17659167 |
| *B. morio* | Agudos | SP | -48.98941944 | -22.46914167 |
| *B. morio* | Santa Rosa do Viterbo | SP | -47.36220000 | -21.47063333 |
| *B. morio* | Ribeirão Preto | SP | -47.82111111 | -21.17659167 |
| *B. morio* | São Sebastião | SP | -45.41052778 | -23.758975 |
| *B. morio* | Brotas | SP | -48.12233700 | -22.281628 |
| *B. pauloensis* | Brasília | DF | -47.92913889 | -15.78016111 |
| *B. pauloensis* | Dores do Rio Preto | ES | -41.84176111 | -20.69324444 |
| *B. pauloensis* | Alto Paraíso de Goiás | GO | -47.53545556 | -14.15404444 |
| *B. pauloensis* | Aiuruoca | MG | -44.60518333 | -21.92950833 |
| *B. pauloensis* | Alto Caparaó | MG | -41.87460833 | -20.43305556 |
| *B. pauloensis* | Baependi | MG | -44.89136667 | -21.95964444 |
| *B. pauloensis* | Belo Horizonte | MG | -43.96573056 | -19.81573056 |
| *B. pauloensis* | Bocaiúva | MG | -43.82121944 | -17.11515556 |
| *B. pauloensis* | Botumirim | MG | -43.00553611 | -16.85218333 |
| *B. pauloensis* | Brasilândia de Minas | MG | -46.01522500 | -17.01936667 |
| *B. pauloensis* | Buenópolis | MG | -44.17281944 | -17.86978056 |
| *B. pauloensis* | Caeté | MG | -43.66981111 | -19.88062222 |
| *B. pauloensis* | Camanducaia | MG | -46.14553333 | -22.75533056 |
| *B. pauloensis* | Diamantina | MG | -43.59553889 | -18.21736389 |
| *B. pauloensis* | Francisco Dumont | MG | -44.24219444 | -17.29459444 |
| *B. pauloensis* | Gonçalves | MG | -45.85440000 | -22.65889444 |
| *B. pauloensis* | Itacambira | MG | -43.31156111 | -17.06403056 |
| *B. pauloensis* | lima Duarte | MG | -43.89994600 | -21.739564 |
| *B. pauloensis* | Nova Lima | MG | -43.84631111 | -19.98759444 |
| *B. pauloensis* | Olhos d`água | MG | -43.57540833 | -17.395775 |
| *B. pauloensis* | São Gonçalo do Rio Preto | MG | -43.39520000 | -18.00658889 |
| *B. pauloensis* | São Roque de Minas | MG | -46.36707500 | -20.232775 |
| *B. pauloensis* | São Roque de Minas | MG | -46.36707500 | -20.232775 |
| *B. pauloensis* | Jaboticatubas | MG | -42.38972222 | -18.68934722 |
| *B. pauloensis* | Serranópolis de Minas | MG | -42.86956389 | -15.81263611 |
| *B. pauloensis* | Uberlândia | MG | -48.26219167 | -18.91130556 |
| *B. pauloensis* | General Carneiro | PR | -51.31723611 | -26.42885556 |
| *B. pauloensis* | Ponta Grossa | PR | -50.16300833 | -25.09454722 |
| *B. pauloensis* | Londrina | PR | -51.17367000 | -23.296075 |
| *B. pauloensis* | Prudentópolis | PR | -50.96893333 | -25.21549722 |
| *B. pauloensis* | Foz do Iguaçu | PR | -53.47734167 | -24.44945556 |
| *B. pauloensis* | Guaratuba | PR | -47.42169444 | -24.08502778 |
| *B. pauloensis* | Matinhos | PR | -48.57835500 | -22.8904 |
| *B. pauloensis* | Otacílio Costa | PR | -50.20030000 | -27.49794 |
| *B. pauloensis* | São Mateus do Sul | PR | -50.42231000 | -26.02515 |
| *B. pauloensis* | Itatiaia | RJ | -44.56094722 | -22.49573056 |
| *B. pauloensis* | Resende | RJ | -44.45670000 | -22.4635 |
| *B. pauloensis* | Caçapava do Sul | RS | -53.48690000 | -30.5414 |
| *B. pauloensis* | Cambará do Sul | RS | -50.14470000 | -29.0478 |
| *B. pauloensis* | Candiota | RS | -53.67919167 | -31.47676944 |
| *B. pauloensis* | Cruz Alta | RS | -53.60654800 | -28.639556 |
| *B. pauloensis* | Guaiba | RS | -51.32500000 | -30.1139 |
| *B. pauloensis* | Igrejinha | RS | -50.78768400 | -29.57444 |
| *B. pauloensis* | Lavras do Sul | RS | -53.89500000 | -30.8131 |
| *B. pauloensis* | Nova Petrópolis | RS | -51.11440000 | -29.3764 |
| *B. pauloensis* | Osório | RS | -50.26970000 | -29.8867 |
| *B. pauloensis* | Pelotas | RS | -52.34250000 | -31.7719 |
| *B. pauloensis* | Porto Alegre | RS | -51.23000000 | -30.0331 |
| *B. pauloensis* | Santana da Boa Vista | RS | -53.11530000 | -30.8719 |
| *B. pauloensis* | Santana de Livramento | RS | -55.53792200 | -30.895154 |
| *B. pauloensis* | São Francisco de Paula | RS | -50.58333300 | -29.448056 |
| *B. pauloensis* | Tenente Portela | RS | -53.75830000 | -27.3711 |
| *B. pauloensis* | Torres | RS | -49.72690000 | -29.3353 |
| *B. pauloensis* | Três Coroas | RS | -50.77167800 | -29.518035 |
| *B. pauloensis* | Viamão | RS | -51.02330000 | -30.0811 |
| *B. pauloensis* | Vacarias | RS | -51.07365000 | -28.54387 |
| *B. pauloensis* | Caxias do Sul | RS | -51.21665000 | -29.12379 |
| *B. pauloensis* | Farroupilha | RS | -51.21679000 | -29.12413 |
| *B. pauloensis* | Painel | SC | -50.10328889 | -27.92495833 |
| *B. pauloensis* | São Joaquim | SC | -49.89290000 | -28.2513 |
| *B. pauloensis* | Atibaia | SP | -46.52933611 | -23.16296667 |
| *B. pauloensis* | Campinas | SP | -47.06080000 | -22.9056 |
| *B. pauloensis* | Franco da Rocha | SP | -46.72478333 | -23.32249167 |
| *B. pauloensis* | Iguape | SP | -47.55530000 | -24.7081 |
| *B. pauloensis* | Itirapina | SP | -47.82293333 | -22.2537 |
| *B. pauloensis* | Luís Antônio | SP | -47.70440000 | -21.555 |
| *B. pauloensis* | Ribeirâo Grande | SP | -48.37152778 | -24.09791944 |
| *B. pauloensis* | Santa Rita do Passa Quatro | SP | -47.47810000 | -21.7103 |
| *B. pauloensis* | São Paulo | SP | -46.63610000 | -23.5475 |
| *B. pauloensis* | Ubatuba | SP | -44.83298056 | -23.36698889 |
| *B. pauloensis* | Apiaí | SP | -48.84320556 | -24.51376667 |
