# Supplementary material for: Comparative phylogeography in the Atlantic forest and Brazilian savannas: pleistocene fluctuations and dispersal shape spatial patterns in two bumblebees
Source: BMC Evol Biol. 2016 Dec 7;16:267. doi: 10.1186/s12862-016-0803-0 (PMC5142330; doi:10.1186/s12862-016-0803-0)
Supplement: Additional file 6: — Heteroplasmy found in the Bombus morio mitochondrial markers (undiscriminated nucleotides) characterized by double peaks, low phred quality scores, and synonymous divergence for the coding regions. (DOCX 981 kb) [file 12862_2016_803_MOESM6_ESM.docx]

**Additional file 6** – Heteroplasmy found in the *B. morio* mitochondrial markers (undiscriminated nucleotides) characterized by double peaks, low phred quality scores and synonymous divergence for the coding regions.
