# Supplementary material for: Comparative phylogeography in the Atlantic forest and Brazilian savannas: pleistocene fluctuations and dispersal shape spatial patterns in two bumblebees
Source: BMC Evol Biol. 2016 Dec 7;16:267. doi: 10.1186/s12862-016-0803-0 (PMC5142330; doi:10.1186/s12862-016-0803-0)
Supplement: Additional file 8: — Mean of likelihood obtained for Bombus morio for each K. (DOCX 37 kb) [file 12862_2016_803_MOESM8_ESM.docx]

**Additional file 8** – Mean of likelihood obtained for *B. morio* for each K
