# Supplementary material for: Comparative phylogeography in the Atlantic forest and Brazilian savannas: pleistocene fluctuations and dispersal shape spatial patterns in two bumblebees
Source: BMC Evol Biol. 2016 Dec 7;16:267. doi: 10.1186/s12862-016-0803-0 (PMC5142330; doi:10.1186/s12862-016-0803-0)
Supplement: Additional file 9: — Delta K obtained for Bombus pauloensis for each K. (DOCX 30 kb) [file 12862_2016_803_MOESM9_ESM.docx]

**Additional file 9**– Delta K obtained for *B. pauloensis* for each K
