# Supplementary material for: Comparative phylogeography in the Atlantic forest and Brazilian savannas: pleistocene fluctuations and dispersal shape spatial patterns in two bumblebees
Source: BMC Evol Biol. 2016 Dec 7;16:267. doi: 10.1186/s12862-016-0803-0 (PMC5142330; doi:10.1186/s12862-016-0803-0)
Supplement: Additional file 11: — Contribution of each climatic variable in the geographic distribution of Bombus morio and B. pauloensis. (DOCX 15 kb) [file 12862_2016_803_MOESM11_ESM.docx]

**Additional file 11** – Contribution of each climatic variable in the geographic distribution of *B. morio* and *B. pauloensis*

| Species | Variable | Contribution (%) |
| --- | --- | --- |
| *B. morio* | Max. temperature of warmest month | 29.1 |
|  | Precipitation of warmest quarter | 24.2 |
|  | Temperature seasonality | 24.2 |
|  | Mean temperature of coldest quarter | 12 |
|  | Precipitation of wettest month | 5.3 |
|  | Isothermality | 3.9 |
|  | Precipitation of driest quarter | 1.3 |
| *B. pauloensis* | Max. temperature of warmest month | 32.4 |
|  | Temperature seasonality | 25.3 |
|  | Precipitation of warmest quarter | 22.3 |
|  | Annual precipitation | 9.4 |
|  | Mean temperature of wettest quarter | 5.8 |
|  | Temperature annual range | 3.1 |
|  | Precipitation of driest month | 1.7 |
| *B. pauloensis* Clade C | Precipitation of warmest quarter | 36.9 |
|  | Precipitation of coldest quarter | 23.7 |
|  | Isothermality | 13.1 |
|  | Max. temperature of warmest month | 9.4 |
|  | Mean temperature of wettest quarter | 8 |
|  | Mean temperature of driest quarter | 6.4 |
|  | Temperature seasonality | 2.4 |
| *B. pauloensis* Clade N | Mean temperature of coldest quarter | 27.9 |
|  | Precipitation of wettest quarter | 20.9 |
|  | Precipitation of wettest quarter | 19.8 |
|  | Precipitation of coldest quarter | 15.9 |
|  | Max. temperature of warmest month | 15.5 |
| *B. pauloensis* Clade S | Precipitation of warmest quarter | 22.1 |
|  | Isothermality | 21.3 |
|  | Mean temperature of driest quarter | 20.4 |
|  | Precipitation of driest month | 17.8 |
|  | Mean temperature of warmest quarter | 16 |
|  | Min. temperature of coldest quarter | 2.4 |
